# Animate Categories Show Higher Cross-Duration Representational Selectivity in Ventral Occipitotemporal Cortex Under Brief Visual Input

**DOI:** 10.3390/brainsci16070668

**Published:** 2026-06-26

**Authors:** Yuying Wang, Xueming Lu

**Affiliations:** 1 School of Psychological and Cognitive Sciences, Peking University, Beijing 100871, China; yywang@stu.pku.edu.cn; 2 School of Psychology, Northeast Normal University, Changchun 130024, China; 3 Jilin Provincial Key Laboratory of Cognitive Neuroscience and Mental Health, Northeast Normal University, Changchun 130024, China

**Keywords:** animacy, animate-category advantage, category information, ventral occipitotemporal cortex, neural representation, fMRI, MVPA

## Abstract

**Highlights:**

**What are the main findings?**

- In the ventral occipitotemporal cortex, animate categories showed higher cross-duration category information than inanimate categories, suggesting that brief-presentation patterns corresponded more selectively to same-category patterns under extended viewing.
- This effect remained after excluding the human-head category and was consistent across ROI-definition strategies and robustness analyses, suggesting that it was not driven solely by human heads or by a particular ROI definition.

**What are the implications of the main findings?**

- Within-category visual homogeneity contributed to cross-duration category information, but the measured feature spaces did not fully account for the animate-category advantage.
- The advantage may reflect both animacy-related representational organization and visual-structural factors, providing a candidate representational account of animate-category processing under limited input.

**Abstract:**

**Background:** The human visual system can extract object-category information from extremely brief visual input, and animate categories often show behavioral advantages over inanimate categories in rapid categorization, visual search, and change-detection tasks. Motivated by these behavioral findings, the present study asked whether, at the representational level, animate categories elicit more category-selective neural patterns than inanimate categories in the ventral visual cortex under brief input. **Methods:** Using fMRI and correlation-based multivoxel pattern analysis (MVPA), we examined whether activity patterns elicited by animate categories in the ventral occipitotemporal cortex under a 33 ms brief-presentation condition corresponded more selectively to same-category patterns under a 500 ms extended-viewing condition than did patterns elicited by inanimate categories. During scanning, participants viewed animate and inanimate stimuli, each comprising four basic-level subcategories, and performed a noise-detection task that did not require explicit category judgments. **Results**: Across multiple ROI-definition strategies, animate categories showed significantly higher cross-duration category information than inanimate categories. This effect also remained significant after excluding the human-head category, which contained human-face information. Stimulus-level image-feature analyses further showed that within-category visual homogeneity explained part of the variance in cross-duration category information, particularly in the full stimulus set that included human heads. However, a composite visual homogeneity index derived from HOG, Gabor, and ResNet50 features did not fully account for the higher cross-duration category information observed for animate categories. **Conclusions**: Overall, these results suggest that, when visual input is highly limited, animate categories elicit VOTC multivoxel patterns that correspond more selectively to same-category patterns under extended viewing.

## 1. Introduction

The human visual system can extract object and scene category information from complex visual input within a remarkably short time. A large body of behavioral evidence has shown that observers can determine whether a natural scene contains animals, people, vehicles, or other categories even when images are presented for only tens of milliseconds [1,2,3]. Such rapid categorization suggests that category information may become available before fine-grained individual identification is completed. Visual object information can be represented at multiple levels, ranging from individual identities to basic-level categories and broader evolutionarily relevant domains [4,5]. Among superordinate distinctions, the animate–inanimate division is considered one of the most commonly used and best-supported category boundaries in visual categorization [2,6,7,8] and has also been proposed as a core dimension in the organization of object knowledge [9,10,11,12].

Previous studies have found that animate stimuli often show processing advantages over inanimate stimuli in rapid go/no-go tasks, saccadic choice, visual search, and change-detection paradigms. Observers can rapidly detect animal targets in complex natural scenes and are more likely to detect changes involving humans or other animals [2,6,8,13,14,15,16]. These findings suggest that the visual system may process animate-related information efficiently across a range of behavioral tasks. At the same time, behavioral responses are the final output of multiple processing stages and may be influenced by visual representation, attentional capture, search strategy, task preparation, decision criteria, and response selection. Thus, behavioral animate advantages alone cannot determine whether such advantages are already reflected in visual cortical representations under limited input. The present study therefore focused on a representational question: whether animate categories show more category-selective neural patterns than inanimate categories in the visual cortex when visual input is highly limited.

fMRI (functional Magnetic Resonance Imaging) studies provide an important framework for addressing this question. Extensive fMRI evidence has shown that the ventral visual pathway, particularly the ventral temporal cortex (VTC), is a core region for object-category representation [17,18]. Within the VTC and adjacent occipitotemporal regions, animacy constitutes a stable and prominent representational dimension [19,20]. Previous studies have shown that faces [21], bodies [22,23], and animate stimuli [12,24] typically elicit stronger responses in the lateral VTC, the lateral occipitotemporal cortex, and regions near the superior temporal sulcus. In contrast, inanimate categories such as scenes, buildings, and tools more often involve the medial VTC, the parahippocampal cortex, and related regions [24,25,26,27]. Together, these findings indicate that animate and inanimate categories show partially dissociable spatial organization in the occipitotemporal visual system, providing a basis for examining category-selective neural patterns under brief input.

Interestingly, some studies suggest that animate-category information may have a distinctive representational distribution in the visual cortex [20,28]. Using encoding models to characterize each voxel’s response preference for different semantic categories, Naselaris et al. [20] found that voxels preferring living-thing information formed a relatively concentrated high-density distribution anterior to retinotopic visual areas, encompassing classical category-selective regions such as face- and body-selective areas and their surrounding cortex. By contrast, voxels preferring nonliving objects were more diffusely distributed and were mainly located in smaller regions along the upper and lower extremes. More recently, EEG and MEG studies examining temporal dynamics have found that animacy-related information can be decoded or distinguished within early time windows after stimulus onset [29,30,31]. This indicates that animate or living-thing information may not emerge only as a semantic classification outcome after extensive processing but may already be available to some extent during early visual processing. These studies suggest, from spatial-organization and temporal-dynamics perspectives, that animacy-related information may be a relatively accessible category dimension in the visual system. However, early decodability primarily indicates when category information becomes distinguishable; it does not by itself show that animate categories have a cross-duration category advantage over inanimate categories. It therefore remains necessary to test whether VOTC multivoxel patterns elicited by animate categories under limited input correspond more selectively to same-category patterns under extended viewing.

To address this question, the present study used an event-related fMRI design with two presentation-duration conditions: target images were presented for 33 ms in the brief-presentation condition and for 500 ms in the long-presentation condition. The 33 ms masked condition was intended to limit sustained visual input, and similar brief masked presentations have been widely used to examine the time course of object detection, categorization, and recognition [4,32]. The 500 ms condition provided relatively extended visible input. Previous fMRI studies of object-category representations have used 500 ms presentations to estimate distributed category patterns in the ventral temporal cortex [17,33], and related MVPA studies have often used presentations of several hundred milliseconds to estimate object-evoked response patterns [19]. Thus, in the present study, the 500 ms condition should be understood as an extended-viewing condition, not as a complete or final visual-semantic representation. The stimuli comprised two superordinate categories, animate and inanimate. Animate subcategories included human heads, human bodies, animal faces, and whole animal bodies; inanimate subcategories included tools, large artifacts, natural objects, and buildings. To reduce the influence of explicit animate–inanimate judgments and category-decision strategies, participants performed a noise-detection task during scanning that did not require judgments of specific categories. We then used correlation-based MVPA in ventral occipitotemporal regions of interest to compute the correlation between each category pattern under the brief-presentation condition and the corresponding category pattern under the long-presentation condition. Category information (CI) was used to quantify cross-duration category selectivity, that is, the extent to which a brief-presentation pattern for a given category corresponded more strongly to the same category than to other categories under extended viewing [17,34]. We predicted that, if patterns elicited by animate categories under brief input correspond more selectively to same-category patterns under extended viewing, animate categories should show higher CI than inanimate categories. Conversely, if this is not the case, the two stimulus classes should not differ in cross-duration category information.

When interpreting a possible animate-category CI advantage, it is also necessary to consider visual-structural differences among the stimulus images. Object-category representations are closely related to shape, low-level image properties, and category attributes [35,36,37]. Low-level image properties can predict response patterns in category-selective regions of the ventral visual pathway [35]. At the same time, studies that explicitly dissociate visual and category-related dimensions have shown that these factors make partially independent but closely related contributions along the visual hierarchy and to VTC animacy organization [36,38]. Proklova et al. [26] further showed that animate–inanimate-category membership can explain part of the neural-pattern differences in the visual cortex even when shape similarity is controlled. These findings suggest that, in studies using natural object images, within-category visual homogeneity may influence correlation-based MVPA results. Thus, if animate categories show higher CI, it is important to evaluate whether this effect can be explained by differences in within-category visual homogeneity. To address this issue, we conducted stimulus-level computational image-feature analyses using HOG, Gabor filter responses, and ResNet50 convolutional features to quantify within-category visual homogeneity for each subcategory and to test the extent to which these visual homogeneity measures accounted for the animate-category CI advantage.

## 2. Methods

### 2.1. Participants

Twenty-one adult participants from Beijing Normal University took part in the experiment (14 females; mean age = 22.81 ± 2.62 years). Subject-level motion quality control used a commonly applied voxel-scale exclusion criterion in task-based fMRI studies. Participants were excluded if their maximum translation exceeded 3 mm in any direction or if their maximum rotation exceeded 3 degrees. This threshold was used to remove data with excessive motion that could introduce spatial misregistration and motion-related artifacts, consistent with prior work on motion effects in fMRI and with task-based fMRI studies using comparable participant-level motion criteria [28,39,40]. Based on this criterion, one participant was excluded because of excessive head motion. Thus, 20 participants were included in the whole-brain analysis. Two additional participants were excluded from the ROI definitions and MVPA because of poor functional-image signal quality: even under a relatively liberal threshold, stable basic visual responses were not observed within the predefined anatomical range. Consequently, 18 participants were included in the ROI definitions and MVPA. All participants were right-handed native Chinese speakers with normal or corrected-to-normal vision, no history of neurological or psychiatric disorders, and no contraindications for MRI scanning. All participants provided written informed consent before the experiment.

### 2.2. Experimental Materials and Procedure

**Figure 1:**
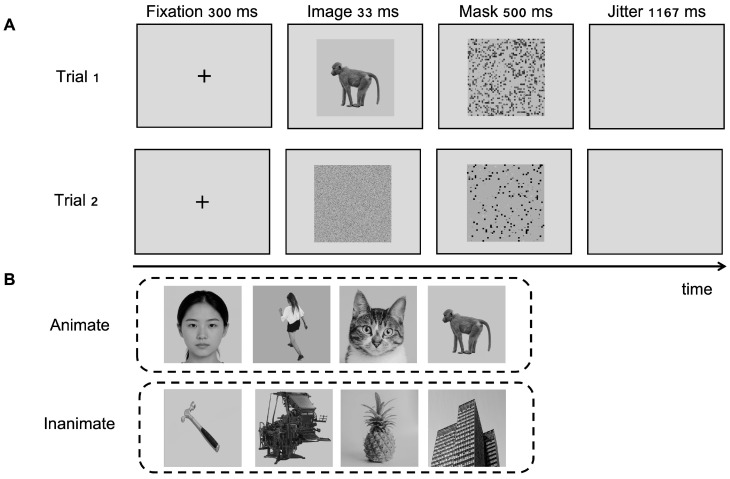
Experimental procedure and stimulus categories. (**A**) Trial structure for the brief-presentation condition. Trial 1 is an object trial, which does not require a response; Trial 2 is a noise trial, which requires a response. The plus sign indicates the fixation point. The long-presentation condition followed the same trial structure, except that the target image was presented for 500 ms and the jitter duration was adjusted accordingly. (**B**) The stimulus set comprised eight object subcategories, grouped into animate and inanimate categories. Animate categories included human heads, human bodies, animal faces, and animal bodies; inanimate categories included tools, large artifacts, natural objects, and buildings. The human face image was generated using OpenAI GPT Image 2.0 for illustrative purposes only. Other images (pineapple, monkey, etc.) are royalty-free images obtained from Unsplash and Pexels.

The experimental stimuli consisted of images photographed by the experimenters or collected from online sources. The stimulus set included eight object subcategories, with 32 images per subcategory, yielding 256 object images in total. These eight subcategories were grouped into two superordinate categories: animate and inanimate. As shown in Figure 1, animate categories included human heads (including faces and hair), human bodies (including faceless whole bodies and body parts), animal faces, and whole animal bodies, whereas inanimate categories included tools (e.g., hammers), large artifacts (e.g., microwave ovens), natural objects (e.g., leaves), and buildings (e.g., temples). All images were preprocessed using MATLAB. First, images were cropped into 400 × 400-pixel grayscale images. Second, non-object information that might appear in the images, such as letters, patterns, or watermarks, was blurred. Third, during stimulus preparation, images from different categories were made as comparable as possible in overall visual quality, object position, and image composition. Finally, images were converted to BMP format for presentation using E-Prime 1.1. To further reduce the influence of low-level visual differences, all processed images were globally matched for luminance in MATLAB (https://www.mathworks.com/, accessed on 24 June 2026) using the SHINE toolbox [41]. However, luminance matching cannot eliminate potential differences among object categories in shape, texture, spatial-frequency structure, part configuration, or within-category visual variability. Therefore, in addition to the main neural analyses, we conducted stimulus-level computational image-feature analyses to quantify visual homogeneity within each subcategory. These analyses are described in Section 2.4.5.

The present study adapted the stimulus-presentation procedure and task rationale of de la Rosa et al. [32], who examined the time course of object detection and categorization and implemented them in an fMRI noise-detection task (Figure 1). The experiment included two presentation-duration conditions: the target was presented for 33 ms in the brief-presentation condition and for 500 ms in the long-presentation condition. The central aim was to assess the extent to which category patterns elicited under brief input corresponded selectively to same-category patterns under extended viewing. The 33-ms masked presentation was used to limit sustained visual input and is consistent with brief masked-presentation procedures commonly used in studies of rapid visual categorization and object recognition [4,32]. The 500 ms presentation provided relatively extended visible input and served as an extended-viewing condition for estimating category-evoked VOTC patterns. Similar object-category fMRI studies have used presentations of several hundred milliseconds or longer to estimate category-related activity patterns [17,19,33]. Importantly, the 500 ms condition was treated as an extended-viewing condition in the present study, not as a complete or final representation. Each participant completed the two presentation-duration conditions in alternating order. Each run contained 160 trials and lasted 6 min 40 s. Of these trials, 128 were object-image trials, and 32 were noise-image trials. In the object-image trials, each subcategory contained 16 trials. Stimulus order was pseudorandomized before the experiment, and the complete stimulus set was divided into two counterbalanced versions so that stimulus assignment to the brief- and long-presentation conditions was balanced across participants.

The trial structure was identical across the two presentation-duration conditions, consisting of a fixation point, a target image, a scrambled-image mask, and a blank-jitter interval. The two conditions differed only in target duration and blank-jitter duration. In the brief-presentation condition, each trial began with a 300 ms fixation point, followed by a 33 ms target image, a 500 ms scrambled-image mask, and a blank-jitter interval. The jitter durations were 1167, 1667, or 2167 ms, yielding total trial durations of 2000, 2500, or 3000 ms. In the long-presentation condition, each trial likewise began with a 300 ms fixation point, followed by a 500 ms target image, a 500 ms scrambled-image mask, and a blank-jitter interval. The jitter durations were 700, 1200, or 1700 ms, again yielding total trial durations of 2000, 2500, or 3000 ms. Thus, the mean trial duration was 2500 ms in both presentation conditions.

The experiment was controlled using E-Prime 1.1 (http://www.pstnet.com, accessed on 24 June 2026), and participants’ right-hand button responses were recorded. To ensure synchronization between stimulus presentation and MRI scanning, the E-Prime program was set to Cumulative running mode, and the PreRelease parameter was set to 100 ms. Before each run, the program waited for a trigger signal from the MRI scanner before starting. During the experiment, participants viewed the projection screen through a mirror mounted on the head coil. The projection screen had a refresh rate of 60 Hz and a spatial resolution of 1024 × 768. Participants performed a noise-detection task. They were instructed to press a button only when a noise image containing no object was presented and to withhold responses on object-image trials. Before the formal scan, participants completed a practice task in the preparation area to ensure that they understood the task requirements.

### 2.3. MRI Data Acquisition

All structural and functional images were acquired at the MRI Center of Beijing Normal University using a 3T Siemens MRI scanner (Siemens Healthineers, Erlangen, Germany). High-resolution structural images were acquired using a sagittal 3D-MPRAGE sequence. The scanning parameters were as follows: TR = 2500 ms, TE = 3.39 ms, flip angle = 7 degrees, matrix size = 256 × 256, number of slices = 144, spatial resolution = 1.33 × 1 × 1 mm, sagittal acquisition orientation, and a scan duration of approximately 6 min. Functional images were acquired using a gradient echo-planar imaging (EPI) sequence to measure the BOLD signal. The scanning parameters were as follows: TR = 2000 ms, TE = 30 ms, flip angle = 90 degrees, matrix size = 64 × 64, voxel size = 3.125 × 3.125 × 3.5 mm, interslice distance = 4.2 mm, number of slices = 33, axial acquisition orientation, and interleaved slice acquisition.

### 2.4. fMRI Data Analysis

#### 2.4.1. Preprocessing

fMRI data were preprocessed and statistically analyzed using SPM12 (https://www.fil.ion.ucl.ac.uk/spm/software/spm12/, accessed on 24 June 2026) implemented in MATLAB 2014a (https://ww2.mathworks.cn/, accessed on 24 June 2026). Preprocessing included removal of the first five TRs from each run, slice-timing correction, three-dimensional head-motion correction, coregistration of functional and structural images, normalization to Montreal Neurological Institute (MNI) space, and resampling of the functional images to 3 × 3 × 3 mm voxels. Whole-brain activation analyses and ROI definitions used data spatially smoothed with a 6 mm full-width at half-maximum (FWHM) Gaussian kernel. In contrast, correlation-based MVPA was performed on unsmoothed data that had undergone all other preprocessing steps to preserve fine-grained multivoxel spatial-pattern information.

#### 2.4.2. Whole-Brain Analysis

First-level general linear models were constructed separately for the brief- and long-presentation conditions. In each model, the eight object subcategories were modeled as predictors of interest, whereas noise images, fixation points, and mask stimuli were included as additional regressors. Six head-motion parameters were also included as nuisance covariates to control for motion-related signals. At the individual level, contrast maps were constructed for each subcategory relative to the noise condition and for animate minus inanimate categories. Each participant’s contrast maps were then entered into second-level random-effects analyses, and group-level activation results were obtained using one-sample *t*-tests. In addition, we directly compared activation for the same subcategory across the two presentation-duration conditions. For each subcategory, paired contrasts were constructed for the long-presentation condition relative to the brief-presentation condition and for the brief-presentation condition relative to the long-presentation condition. These duration-related activation differences were tested at the group level using paired-samples *t*-tests. Whole-brain statistical maps were first thresholded at an uncorrected voxel-level threshold of *p* < 0.001 as the initial cluster-forming threshold, followed by cluster-level FWE correction with a significance threshold of *p* < 0.05. Cluster-size correction was performed using REST V1.8_130615 (https://rfmri.org/REST, accessed on 24 June 2026) [42]. AlphaSim Monte Carlo simulation, with a voxel-wise threshold of *p* < 0.001, a Gaussian smoothing kernel of 6 mm FWHM, a cluster connection radius of 5 mm, 10,000 simulations, and correction within the analysis mask. Under these parameters, clusters of at least 13 voxels corresponded to a corrected cluster-level alpha of <0.05. Peak coordinates of significant activation clusters are reported in MNI space in Supplementary Tables S1–S3.

#### 2.4.3. Region-of-Interest (ROI) Definition

Because the ROI-definition strategy can influence MVPA results and functionally defined ROIs may introduce voxel-selection bias, the present study used three ROI-definition strategies within the same ventral occipitotemporal anatomical extent: participant-specific Joint-ROIs, a group-level Group-ROI, and a purely anatomical Anatomical-ROI. These ROIs should not be interpreted as three independent anatomical regions. Rather, they are analysis masks defined within the same VOTC territory using different criteria and may therefore spatially overlap.

The first strategy was the participant-specific Joint-ROI. This ROI was defined using spatially smoothed functional data from the long-presentation condition. For each participant, contrast maps were first constructed in the first-level model for each of the eight subcategories relative to the noise condition. The search space was then restricted to a bilateral ventral occipitotemporal anatomical mask defined from the AAL template, including the parahippocampal gyrus, inferior occipital gyrus, fusiform gyrus, and inferior temporal gyrus. These regions cover occipitotemporal areas previously associated with distributed object representations and with face-, body-, and scene-related processing [17,22,23,25]. For each subcategory, the local peak for the subcategory-versus-noise contrast was identified within this anatomical mask, and a 9 × 9 × 9-voxel search cube was centered on that peak [27,43]. Within each search cube, the top-N voxels were selected according to activation strength to form a category-specific functional mask. This procedure restricted candidate voxels to a local region around each category peak but did not additionally require the selected top-N voxels to form a single contiguous cluster. The masks for the eight subcategories were then combined to form the participant-specific Joint-ROI. The main analysis used the top 100 voxels per subcategory. To assess whether the results depended on this voxel-number choice, we repeated the ROI definitions in supplementary analyses using the top 50, 75, 125, 150, 175, and 200 voxels. This category-balanced procedure reduced the possibility that the ROI was driven primarily by a single strongly activated category, but it did not make the ROI fully independent of the experimental stimuli. Therefore, we also used the Group-ROI and Anatomical-ROI as complementary tests.

The second strategy was the group-level Group-ROI, which was used to test whether the main results were also present in VOTC visual-response regions shared across participants and did not depend solely on individual peak selection. This ROI was also defined using spatially smoothed functional data from the long-presentation condition. For each participant, we first constructed the contrast of all object categories relative to the noise condition. Individual contrast maps were then entered into a group-level one-sample *t*-test to identify object-evoked visual-response regions that were consistent across participants. Finally, the group-level activation map was constrained by the same AAL-based ventral occipitotemporal anatomical mask used for the Joint-ROI, yielding the Group-ROI.

The third strategy was the Anatomical-ROI. This ROI did not use any functional activation information and was defined by combining the bilateral parahippocampal gyrus, inferior occipital gyrus, fusiform gyrus, and inferior temporal gyrus from the AAL template. The Anatomical-ROI therefore provided a conservative test independent of stimulus-evoked activation and functional voxel selection, allowing us to evaluate whether the main effect could still be observed within a non-functionally defined VOTC anatomical extent.

#### 2.4.4. Cross-Duration MVPA

To examine how closely object-category representations under the brief-presentation condition resembled the corresponding category representations under the long-presentation condition, we used correlation-based MVPA. By computing the difference between within-category and between-category correlations, this method provides an independent cross-condition representational selectivity index for each category and has been widely used to quantify the extent to which category-specific neural patterns are preserved across different processing conditions [17,28,34,44,45]. In the present study, this analysis quantified the extent to which category patterns in the brief-presentation condition selectively matched same-category patterns in the long-presentation condition.

For each participant and each ROI-definition strategy, voxel patterns for the eight object subcategories were extracted separately from the brief- and long-presentation conditions. The pattern for each category consisted of the *t*-value map for that category relative to the noise condition. The voxel pattern for each category in the brief-presentation condition was then correlated, using Pearson correlations, with the voxel patterns for all eight categories in the long-presentation condition, producing an 8 × 8 correlation matrix. This correlation matrix contained two types of correlations. The first was the within-category correlation, namely the correlation between a category in the brief-presentation condition and the same category in the long-presentation condition. For example, this included the correlation between the human-head pattern under the brief-presentation condition and the human-head pattern under the long-presentation condition. The second was the between-category correlation, namely the correlation between a category in the brief-presentation condition and other categories in the long-presentation condition. For example, this included the correlation between the human-head pattern under the brief-presentation condition and the patterns for human bodies, animal faces, or tools under the long-presentation condition. All correlation coefficients were Fisher *z*-transformed. Category information was then calculated for each category according to the metric used by Peelen and Kastner [34]. Category information was defined as the difference between the within-category correlation for a given category and the average of its between-category correlations. The formula was

$${\mathrm{CI}}_{i}=Z\left(r_{i,i}\right)\;-\;\frac{1}{n\;-\;1}\sum_{j\neq i}Z\left(r_{i,j}\right)$$

where ${\mathrm{CI}}_{i}$ denotes the information for category *i*; $Z\left(r_{i,i}\right)$ denotes the Fisher *z*-transformed correlation coefficient between category *i* in the brief-presentation condition and the same category *i* in the long-presentation condition; $Z\left(r_{i,j}\right)$ denotes the Fisher *z*-transformed correlation coefficient between category *i* in the brief-presentation condition and another category *j* in the long-presentation condition; and n denotes the total number of categories, which was 8 in the present study.

For each participant and each ROI-definition strategy, we first obtained CI values for the eight subcategories. A repeated-measures ANOVA with subcategory as a within-participant factor was then conducted to test whether cross-duration representational selectivity differed across subcategories; post hoc comparisons were corrected for multiple comparisons using the Bonferroni method when appropriate. Based on the main hypothesis, we further performed planned paired comparisons by computing the mean CI across the four animate subcategories and the four inanimate subcategories separately and comparing them using paired-samples *t*-tests. In addition to reporting paired *t*-tests, *p* values, and Cohen’s dz, we estimated 95% confidence intervals for the animacy advantage using participant-level bootstrap resampling and used sign-flipping permutation tests to evaluate whether the paired differences differed significantly from zero. To test whether the main effect was driven primarily by the human-head category, which contained human-face information, we repeated the animate–inanimate comparison after excluding the human-head category; that is, we compared the mean CI of the remaining three animate subcategories with the mean CI of the four inanimate subcategories. Finally, to assess whether the results depended on individual participants, we conducted leave-one-subject-out stability analyses. In each iteration, one participant was excluded, and the animacy advantage was recalculated using the remaining 17 participants; we then recorded whether the effect direction remained consistent.

Finally, although the scanner task did not require explicit animate–inanimate categorization and therefore could not directly test the relationship between rapid categorization behavior and the neural index, we conducted exploratory brain-behavior correlation analyses using performance in the scanner noise-detection task. For each participant, we extracted overall accuracy, noise-detection accuracy, and reaction time for correctly detected noise trials separately for the two presentation-duration conditions. These behavioral measures were then correlated, using Spearman correlations, with the animacy CI advantage in each of the three ROI-definition strategies. The animacy CI advantage was defined as the mean CI for animate categories minus the mean CI for inanimate categories.

#### 2.4.5. Stimulus-Level Visual Homogeneity Analysis

To test whether the animacy effect in cross-duration category information could be influenced by differences in visual homogeneity across stimulus categories, we conducted computational image-feature analyses on the 256 preprocessed stimulus images that were actually presented in the experiment. Three types of computable image features were extracted.

First, a histogram of oriented gradients (HOG) features was used to characterize local edge orientation, contour distribution, and shape structure [46]. Second, Gabor filter responses were used to characterize energy distributions across spatial frequencies and orientations, as well as texture-related mid-level visual statistics [47]. Third, we extracted convolutional-layer features from a ResNet50 model pretrained on ImageNet [48,49]. Compared with hand-crafted features such as HOG and Gabor responses, convolutional neural networks pretrained on large-scale image-classification tasks provide hierarchical visual features that capture more complex local shapes, texture combinations, and part-like structures [1,49,50]. We selected layers 1–3 of ResNet50 as supplementary image-feature spaces to approximate local texture, shape, and part-structure information. Compared with the final classification layer, these early-to-intermediate convolutional layers are better suited for characterizing image appearance, texture, local shape, and part structure. Importantly, deep convolutional features should not be treated as purely low-level visual features, nor can they fully separate visual and semantic information [51]. Previous studies have shown that category dimensions such as animacy are correlated with low- and mid-level visual features but also show neural organization beyond low-level visual features [31,37]. Moreover, deep neural networks and the VTC do not always weight animal appearance and true animal category in the same way [28]. In the present study, these features were used only to evaluate whether the measured image structure could account for the animacy effect observed in neural CI.

Before feature extraction, all images were converted to grayscale and resized according to the input requirements of each feature model. HOG, Gabor, and ResNet50 layers 1–3 yielded feature vectors of 1764, 80, 256, 512, and 1024 dimensions, respectively. Detailed parameters and implementation procedures are reported in Supplementary Methods S1. For each feature space, feature dimensions were first z-score standardized, and pairwise cosine similarity was then computed among all 256 images, yielding a 256 × 256 image-similarity matrix. For each subcategory, we extracted the 496 non-redundant image pairs among its 32 images and averaged their cosine similarities. This average within-subcategory similarity served as the visual homogeneity index for that subcategory in the corresponding feature space.

To test whether animate subcategories were, overall, more visually homogeneous than inanimate subcategories, we treated the eight subcategories as the statistical units and compared the mean within-category similarity of the four animate subcategories with that of the four inanimate subcategories. Statistical significance was assessed using two-sided label-permutation tests. Within each feature space, the animate–inanimate labels of the eight subcategories were randomly permuted, and the difference between the two mean visual homogeneity values was recalculated. This procedure was repeated 10,000 times to construct the null distribution. Given the special visual structure and neural relevance of the human-head category, we repeated the same analysis after excluding human heads.

Finally, to test whether within-category visual homogeneity could account for the animacy effect in cross-duration CI, we fitted linear regression models. Each observation corresponded to the CI value for one participant, one ROI-definition strategy, and one subcategory. The baseline model included animacy contrast coding and subject fixed effects, with animacy coded as animate = 0.5 and inanimate = −0.5. The visual-control model additionally included a category-level visual homogeneity measure. Because the five visual homogeneity indices derived from HOG, Gabor, and ResNet50 layers 1–3 all characterized within-category image similarity and were highly correlated, including them simultaneously in the same model could introduce multicollinearity. Therefore, we performed principal component analysis (PCA) of the five standardized visual homogeneity indices across the eight subcategories and used the first principal component as a composite visual homogeneity index, termed Visual homogeneity PC1. All models included subject fixed effects to control for individual differences in the overall CI level. Standard errors were clustered by subject to account for non-independence among category observations from the same participant. Full model specifications and regression outputs are reported in Supplementary Table S5.

## 3. Results

Participants’ mean accuracy in the noise-detection task was 96.3% (SD = 3.77%), indicating that participants could reliably distinguish noise images from object images during the task.

### 3.1. Whole-Brain Analysis Results

**Figure 2:**
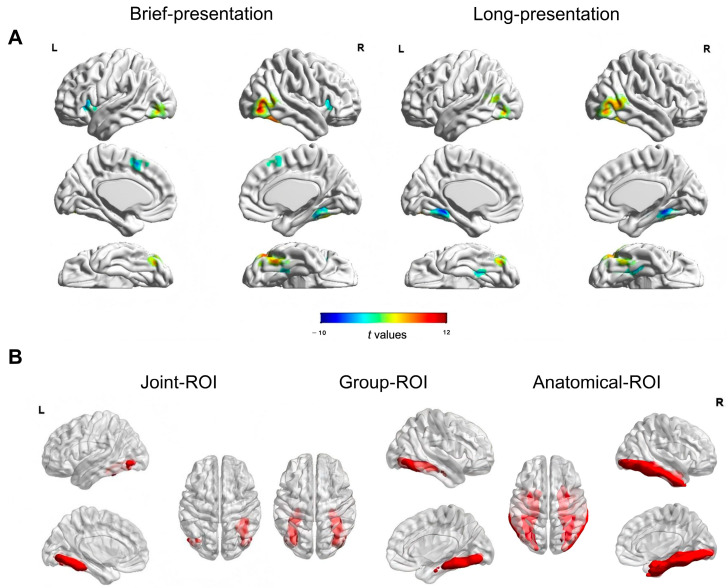
Whole-brain activation maps and ROI definitions. (**A**) Whole-brain activation maps for the eight subcategories under brief (33 ms) and long (500 ms) presentation conditions. The color scale indicates t-values. (**B**) Illustration of the three ROI-definition strategies within the ventral occipitotemporal cortex: participant-specific Joint-ROIs, the group-level Group-ROI, and the Anatomical-ROI. L and R indicate the left and right hemispheres, respectively. Brain maps were visualized using BrainNet Viewer 1.63 [52].

The whole-brain analysis primarily compared differences in brain activity elicited by animate and inanimate objects in order to examine the activation distributions of the two stimulus classes in the ventral visual pathway and related cortical regions. Whole-brain activation patterns for the brief- and long-presentation conditions are shown in Figure 2A. The corresponding significant clusters, including direct comparisons between the two presentation-duration conditions for each subcategory, are reported in Supplementary Tables S1–S3.

In the brief-presentation condition, activation elicited by animate relative to inanimate stimuli was mainly distributed in the bilateral lateral fusiform gyrus, the lateral occipitotemporal cortex, and regions near the right superior temporal sulcus. By contrast, activation elicited by inanimate relative to animate stimuli was biased toward medial regions of the ventral temporal cortex, especially the right parahippocampal gyrus and adjacent regions. In addition, inanimate stimuli showed significant activation in the supplementary motor area and parts of the prefrontal cortex. In the long-presentation condition, activation elicited by animate relative to inanimate stimuli was mainly distributed in the bilateral fusiform gyrus, the bilateral lateral occipitotemporal cortex, regions near the bilateral superior temporal sulcus, and the inferior occipital gyrus. Activation for inanimate relative to animate stimuli was mainly distributed in the medial fusiform gyrus, the parahippocampal gyrus, and adjacent regions.

Across the two presentation-duration conditions, animate activation was mainly located in the lateral occipitotemporal cortex and lateral fusiform regions under both brief and long presentations, whereas inanimate activation was biased toward the medial regions of the ventral temporal cortex. We further performed direct paired comparisons between the two presentation-duration conditions. At the superordinate-category level, neither the animate nor the inanimate contrast showed significant clusters for long presentation greater than brief presentation or for brief presentation greater than long presentation. At the subcategory level, stronger activation in the long-presentation condition was observed mainly for several inanimate subcategories, including large artifacts, natural objects, and buildings, involving the middle and inferior occipital regions, the fusiform cortex, and the parahippocampal cortex. In contrast, stronger activation for brief than long presentation was observed only for the human-body versus noise contrast, with a cluster in the right-middle frontal gyrus. Overall, the whole-brain analysis showed that animate and inanimate categories exhibited relatively dissociable activation distributions under both brief- and long-presentation conditions.

### 3.2. ROI Pattern Analysis Results

The three ROI masks are shown in Figure 2B. All three ROIs were located within the ventral occipitotemporal cortex, but they should not be interpreted as independent anatomical regions. Rather, they represent different ROI-definition strategies applied within the same VOTC anatomical extent.

We first tested whether cross-duration category information differed among the eight object subcategories.

**Figure 3:**
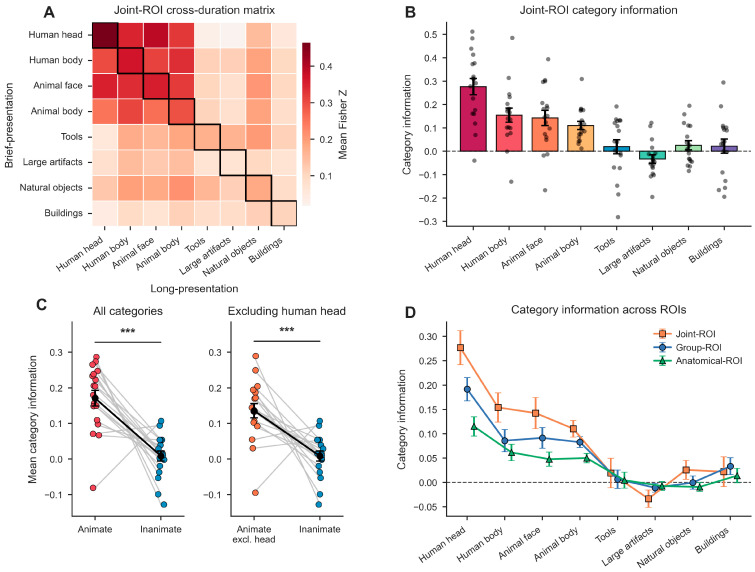
Cross-duration category information. (**A**) Cross-duration pattern-correlation matrix in the Joint-ROI. Each cell represents the mean Pearson correlation coefficient after Fisher’s z-transformation. Black boxes indicate within-category correlations between the two duration conditions. (**B**) Category information values for the eight subcategories in the Joint-ROI. The gray circles represent individual participants, the bars represent the group means, and the error bars represent the standard errors. (**C**) Mean CI for animate and inanimate categories in the full stimulus set and after excluding the human-head category. The left panel shows the comparison, including all animate and inanimate subcategories, and the right panel shows the comparison after excluding the human-head category. Gray lines connect paired data from the same participant, black solid lines connect the group means, and horizontal significance bars with asterisks indicate significant paired comparisons. *** *p* < 0.001. (**D**) Subcategory-level CI values across the Joint-ROI, Group-ROI, and Anatomical-ROI. Joint-ROI results are shown for the top-100 voxel threshold; additional top-N sensitivity results are reported in Supplementary Figures S3–S5 and Supplementary Tables S9–S10. The dashed gray horizontal line indicates zero category information.

Repeated-measures ANOVAs revealed significant category effects in all three ROI-definition strategies. The effects were significant in the Joint-ROI (*F*(7,119) = 12.90, *p* < 0.001, *η*_p_^2^ = 0.431); the Group-ROI (*F*(7,119) = 13.40, *p* < 0.001, *η*_p_^2^ = 0.441); and the Anatomical-ROI (*F*(7,119) = 8.41, *p* < 0.001, *η*_p_^2^ = 0.331). Post hoc comparisons indicated that human heads, human bodies, and animal faces showed significantly higher CI values than several inanimate subcategories. The complete pairwise-comparison results are reported in Supplementary Table S4. The Joint-ROI results are shown in Figure 3, and the corresponding Group-ROI and Anatomical-ROI results are shown in Supplementary Figures S1 and S2.

Planned comparisons further showed that mean CI was significantly higher for animate than for inanimate categories in all three ROI-definition strategies: Joint-ROI—*t*(17) = 4.87, *p* < 0.001, Cohen’s *d*_z_ = 1.15, 95% CI [0.09, 0.23]; Group-ROI—*t*(17) = 5.55, *p* < 0.001, Cohen’s *d*_z_ = 1.31, 95% CI [0.07, 0.15]; and Anatomical-ROI—*t*(17) = 4.29, *p* < 0.001, Cohen’s *d*_z_ = 1.01, 95% CI [0.04, 0.10]. Because the human-head category contained face-related information, we repeated the analyses after excluding this category. The remaining seven subcategories still showed significant category effects in the Joint-ROI (*F*(6,102) = 6.96, *p* < 0.001, *η*_p_^2^ = 0.290); Group-ROI (*F*(6,102) = 6.39, *p* < 0.001, *η*_p_^2^ = 0.273); and Anatomical-ROI (F(6,102) = 4.56, *p* < 0.001, *η*_p_^2^ = 0.212). Critically, mean CI for the remaining animate categories remained significantly higher than mean CI for inanimate categories in all three ROIs: Joint-ROI—*t*(17) = 4.02, *p* < 0.001, Cohen’s *d*_z_ = 0.95, 95% CI [0.06, 0.19]; Group-ROI*—t*(17) = 3.98, *p* < 0.001, Cohen’s *d*_z_ = 0.94, 95% CI [0.04, 0.12]; and Anatomical-ROI—*t*(17) = 3.40, *p* = 0.003, Cohen’s *d*_z_ = 0.80, 95% CI [0.02, 0.09]. These planned-comparison results are summarized in Table 1.

We next examined whether the animacy CI advantage depended on any single animate subcategory. Across the three ROI-definition strategies, each animate subcategory showed significantly higher CI than the mean CI of the inanimate categories after false-discovery-rate correction (all FDR-corrected *p*s ≤ 0.027). In addition, leave-one-animate-subcategory-out analyses showed that the animate–inanimate CI difference remained significant after removing each animate subcategory in turn (all FDR-corrected *p*s ≤ 0.003). The full results are reported in Supplementary Tables S6–S8.

We also tested the robustness of the animacy CI advantage across voxel-selection thresholds and participants. In the Joint-ROI, the full eight-category analysis remained significant across the top-N thresholds from 50 to 200 voxels, with mean animate–inanimate CI differences ranging from 0.14 to 0.19, *t*s(17) ≥ 4.57, *p*s < 0.001, and Cohen’s *d*_z_ = 1.08–1.18. After excluding the human-head category, the effect also remained significant across the same top-N range, with mean differences ranging from 0.11 to 0.15, *t*s(17) ≥ 3.80, *p*s < 0.001, and Cohen’s *d*_z_ = 0.90–0.99. Across the three ROI-definition strategies and top-N thresholds, leave-one-subject-out analyses consistently showed a higher mean CI for animate than for inanimate categories. The full top-N sensitivity, bootstrap, permutation, and leave-one-subject-out results are reported in Supplementary Tables S9 and S10 and Supplementary Figures S3–S5.

Finally, exploratory brain–behavior correlation analyses did not reveal reliable participant-level associations between scanner-task performance and the neural animacy CI advantage. In the brief-presentation condition, overall accuracy, noise-detection accuracy, and reaction time for correctly detected noise trials were not significantly correlated with the animacy CI advantage in any of the three ROI-definition strategies (uncorrected *p*s ≥ 0.247; FDR-corrected *p*s ≥ 0.714). The same pattern was observed in the long-presentation condition (uncorrected *p*s ≥ 0.437; FDR-corrected *p*s = 0.991). These results are reported in Supplementary Tables S11–S15.

### 3.3. Visual Homogeneity Analysis

**Figure 4:**
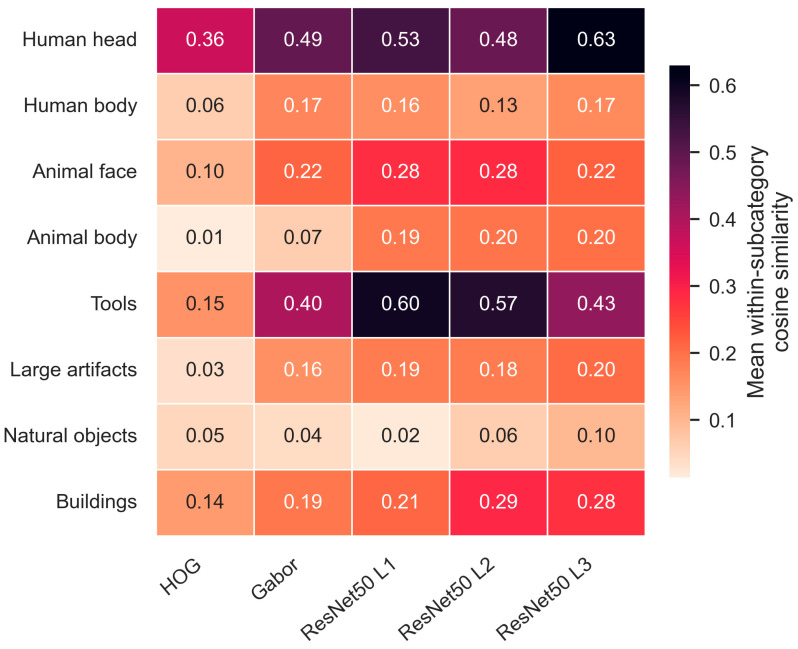
Within-subcategory visual similarity across image-feature spaces. The heat map shows the mean within-subcategory cosine similarity for the eight stimulus subcategories across five image-feature spaces. Each cell represents the mean cosine similarity across all non-redundant image pairs within the same subcategory. HOG features primarily characterize local edge orientation and contour structure, Gabor filter responses primarily characterize the spatial-frequency and orientation energy, and the ResNet50 layers 1–3 features provide complementary measures of local texture, shape, and part-structure information. The color intensity indicates the magnitude of the within-subcategory visual similarity, with higher values indicating greater visual homogeneity in the corresponding feature space. The full numerical values are reported in Supplementary Table S16, and the image-similarity matrices for each feature space are shown in Supplementary Figure S6.

To evaluate whether stimulus-level visual structure could account for the animacy CI advantage, we quantified within-subcategory visual homogeneity using the HOG, Gabor, and ResNet50 feature spaces (Figure 4). The resulting pattern suggested that visual homogeneity varied substantially across individual subcategories rather than showing a stable superordinate animate–inanimate difference. Human heads showed high within-subcategory similarity across all five feature spaces, whereas animal bodies showed relatively low similarity, especially in the HOG feature space. Tools also showed high similarity in several ResNet50 layers. Thus, high visual homogeneity was not unique to animate categories.

Permutation tests comparing mean visual homogeneity between animate and inanimate categories showed no significant animate–inanimate difference in either the full stimulus set or the stimulus set excluding human heads, across the HOG, Gabor, and early-to-intermediate ResNet50 feature spaces. Thus, although individual subcategories differed in visual homogeneity, we found no stable evidence that animate categories were globally more visually homogeneous than inanimate categories. These results are summarized in Table 2.

To obtain a composite visual homogeneity index and to avoid entering multiple highly correlated feature measures into the same regression model, we performed a principal component analysis on the five visual homogeneity measures. The first principal component, hereafter referred to as Visual homogeneity PC1, explained 91.01% of the total variance and had positive loadings on HOG, Gabor, and ResNet50 layers 1–3 features. The full loading values are reported in Supplementary Table S17. Thus, higher PC1 scores indicated greater within-subcategory visual similarity across multiple feature spaces. Human heads had the highest PC1 score (4.08), and tools also had a relatively high PC1 score (2.79), further indicating that high visual homogeneity was not unique to animate categories. The subcategory-level PC1 scores are reported in Supplementary Table S18. Visual homogeneity PC1 was used as the composite visual homogeneity index in the subsequent regression analyses.

The regression results are reported in Table 3. In the full eight-category stimulus set, Visual homogeneity PC1 significantly predicted CI in all three ROI-definition strategies: Joint-ROI—*β* = 0.04, *SE* = 0.01, *t* = 3.53, *p* < 0.001, 95% CI [0.02, 0.05]; Group-ROI—*β* = 0.03, *SE* = 0.01, *t* = 3.31, *p* < 0.001, 95% CI [0.01, 0.04]; and Anatomical-ROI—*β* = 0.02, *SE* = 0.01, *t* = 2.82, *p* = 0.005, 95% CI [0.01, 0.03]. This indicates that within-subcategory visual homogeneity explains part of the variance in CI. Importantly, after Visual homogeneity PC1 was added to the model, the animacy contrast remained a significant predictor of CI: Joint-ROI—*β* = 0.15, *SE* = 0.04, *t* = 4.36, *p* < 0.001, 95% CI [0.09, 0.22]; Group-ROI—*β* = 0.10, *SE* = 0.02, *t* = 4.86, *p* < 0.001, 95% CI [0.06, 0.14]; and Anatomical-ROI—*β* = 0.06, *SE* = 0.02, *t* = 3.84, *p* < 0.001, 95% CI [0.03, 0.10]. Thus, the composite visual homogeneity index measured here did not fully account for the higher CI observed for animate than for inanimate categories.

After the human-head category was excluded, Visual homogeneity PC1 no longer significantly predicted CI in any ROI-definition strategy: Joint-ROI—*β* = 0.01, *SE* = 0.02, *t* = 0.38, *p* = 0.700, 95% CI [−0.03, 0.04]; Group-ROI—*β* = 0.01, *SE* = 0.01, *t* = 0.48, *p* = 0.629, 95% CI [−0.02, 0.03]; and Anatomical-ROI—*β* = 0.01, *SE* = 0.01, *t* = 0.56, *p* = 0.575, 95% CI [−0.01, 0.02]. In the same models, the animacy contrast remained significant: Joint-ROI*—β* = 0.13, *SE* = 0.04, *t* = 3.73, *p* < 0.001, 95% CI [0.06, 0.20]; Group-ROI—*β* = 0.08, *SE* = 0.02, *t* = 3.65, *p* < 0.001, 95% CI [0.04, 0.13]; and Anatomical-ROI*—β* = 0.05, *SE* = 0.02, *t* = 3.09, *p* = 0.002, 95% CI [0.02, 0.09]. These results suggest that the human-head category contributed substantially to the association between visual homogeneity and CI in the full stimulus set, but that the animacy CI advantage did not entirely depend on the human-head category.

Overall, the stimulus-level visual homogeneity analyses showed that the present stimulus set contained subcategory-level differences in visual structure and that the composite visual homogeneity index explained part of the variance in CI, especially in the full stimulus set that included human heads. However, animate categories did not show a stable overall visual homogeneity advantage in the measured HOG, Gabor, and ResNet50 layers 1–layer 3 feature spaces. Moreover, the animacy CI advantage remained significant after controlling for Visual homogeneity PC1. Thus, the present results do not support the interpretation that the animacy CI advantage was driven solely by greater within-category visual homogeneity.

## 4. Discussion

The present study examined whether animate categories show higher cross-duration category information than inanimate categories in the ventral occipitotemporal cortex under brief visual input and further assessed whether this effect could be accounted for by within-category visual homogeneity in the stimulus set. The main results showed that multivoxel patterns elicited by animate categories under the 33 ms brief-presentation condition corresponded more selectively to same-category patterns under the 500 ms long-presentation condition than did patterns elicited by inanimate categories. This effect was observed across participant-specific Joint-ROIs, the group-level Group-ROI, and the activation-independent Anatomical-ROI. It remained significant after excluding the human-head category and was consistent across top-N voxel thresholds and leave-one-subject-out analyses. Stimulus-level image-feature analyses further showed that within-category visual homogeneity explained part of the variance in cross-duration category information, especially in the full stimulus set that included human heads. However, animate categories did not show a stable overall visual homogeneity advantage in the measured HOG, Gabor, and ResNet50 feature spaces, and the animacy CI advantage remained significant after controlling for the composite visual homogeneity index. These findings suggest that within-category visual homogeneity contributed to CI, but that the measured low- to mid-level visual homogeneity indices did not fully account for the observed animate-category advantage.

### 4.1. Cross-Duration Category Selectivity for Animate Categories

The whole-brain activation results were broadly consistent with previous observations of category-related organization in the occipitotemporal cortex. Relative to inanimate categories, animate categories involved more lateral fusiform, lateral occipitotemporal, and superior temporal regions, whereas inanimate categories involved more medial ventral temporal and parahippocampal regions [21,23,24,25,26,27]. Beyond these univariate differences in activation location, the central finding of the present study was that animate categories showed higher category selectivity in cross-duration multivoxel pattern analyses. Specifically, activity patterns elicited by animate categories under brief presentation corresponded more strongly to same-category patterns under long presentation than to patterns of other categories, resulting in higher CI values.

This finding extends previous work on animacy-related organization in the ventral visual pathway. Prior fMRI and MVPA studies have suggested that animacy is an important dimension of object representation in the high-level visual cortex [20,26,27]. The present results suggest that, even when visual input is brief, activity patterns elicited by animate categories in the VOTC show stronger selective correspondence with same-category patterns under longer viewing. This effect should be interpreted as a difference in category selectivity within the distributed representational space of the ventral occipitotemporal cortex, rather than as evidence that animate categories are processed by an independent neural module. Neuropsychological double dissociations between animate and inanimate recognition [11,53] indicate some degree of separable organization within the visual-semantic system, but this separability is more plausibly understood as differentiated feature-structure organization within a distributed system [12], rather than as fully independent processing channels.

The effect did not depend on a single ROI-definition strategy. The participant-specific Joint-ROI was designed to increase sensitivity to individual variability in VOTC functional organization, but because its definition relied on stimulus-evoked functional information, it was important to evaluate whether the result depended on functional voxel selection. The Anatomical-ROI added in the revised analysis was defined entirely from AAL anatomical regions and did not use stimulus-evoked activation information. The animacy CI advantage was still observed in this ROI. The effect was also present in the Group-ROI and remained stable across different top-N voxel thresholds in the Joint-ROI. These results reduce the likelihood that the main finding was driven by a particular ROI-definition procedure or by a specific voxel-number threshold.

Another issue concerns whether the animate-category advantage was primarily driven by the human-head category. Face information elicits strong selective responses in the ventral visual system [21], and the CI advantage observed for the full animate set could therefore be questioned as a byproduct of human-head- or face-related responses. After the human-head category was excluded, the mean CI for the remaining animate categories remained significantly higher than that for inanimate categories. In addition, leave-one-animate-subcategory-out analyses showed that the animacy CI advantage remained significant after each animate subcategory was removed in turn. These results suggest that the present effect was not entirely dependent on a single animate subcategory. Nevertheless, animate categories are not a homogeneous set. Human heads, human bodies, animal faces, and animal bodies differ in faceness, bodyness, human similarity, and biological-form structure [33,37,38,54]. Future studies should manipulate these dimensions more systematically to separate the contributions of faces, bodies, biological form, and animacy per se to cross-duration CI.

The animacy CI advantage may also be considered in relation to the overrepresentation of animal appearance in the VTC. Bracci et al. [28] reported that VTC activity patterns were strongly influenced by animal appearance: inanimate objects with animal-like appearance were represented more similarly to real animals than to typical inanimate objects. They suggested that animal information may be overrepresented relative to object information in the VTC, such that objects carrying animal-like visual cues are partly represented in an animal-like manner. From a related perspective, Bracci et al. [28] noted that a bias toward animal cues may be adaptive because the cost of mistaking a dangerous animal for an inanimate object may be greater than the reverse error. The present findings are consistent with the possibility that, under limited input, animacy-related cues may more readily support stable category correspondence within the distributed VOTC representational space. This interpretation is also compatible with domain-specific accounts and with animal-monitoring hypotheses. Domain-specific accounts emphasize partially separable organization for living things and artifacts within the visual-semantic system [11,12,53], whereas animal-monitoring studies suggest that animate targets such as humans and other animals may receive priority in attention and change detection [13,16].

### 4.2. Contribution of Visual Homogeneity to Animate-Category Selectivity

The interpretation of the animacy CI advantage must take into account visual-structural differences in natural image stimuli. Animate and inanimate categories typically differ not only in semantic properties but also in shape, texture, spatial-frequency structure, curvature, part configuration, symmetry, and within-category variability. This issue is particularly relevant for correlation-based MVPA because stimuli with higher within-category visual homogeneity may elicit more consistent neural patterns and thereby increase cross-duration pattern similarity. The stimulus-level analyses in the present study support this cautious interpretation. The composite visual homogeneity index significantly predicted CI, indicating that within-category visual homogeneity explained part of the variance in neural category information. Human heads, in particular, showed high visual homogeneity across several feature spaces and contributed substantially to the association between visual homogeneity and CI in the full stimulus set. Thus, the animacy CI advantage should not be interpreted as a purely semantic effect without considering visual structure.

At the same time, the present results do not support a simple visual homogeneity account. First, high visual homogeneity was not a shared property of all animate subcategories. Human heads showed high within-category similarity across several feature spaces, but human bodies and animal bodies did not show consistently high visual homogeneity, and some inanimate subcategories, such as tools, also showed relatively high visual similarity. Second, permutation tests using subcategories as the unit of analysis did not provide reliable evidence that animate categories were globally more visually homogeneous than inanimate categories in the HOG, Gabor, and ResNet50 layer feature spaces. Third, after the human-head category was excluded, the remaining animate categories were not more visually homogeneous than the inanimate categories in these feature spaces, yet the animacy CI advantage remained significant. Finally, in regression models, the animacy coefficient remained significant after the composite visual homogeneity index was included. Taken together, these findings suggest that the observed animacy CI advantage was not fully explained by the within-category visual homogeneity measures used in the present study.

Nevertheless, the present visual homogeneity control should be understood as a limited model-based control, not as a complete removal of all visual-semantic confounds. Previous work has shown that low- and mid-level visual attributes are themselves related to animacy judgments [37], whereas the neural dynamics of animacy processing cannot be fully reduced to low-level visual features [31]. Bracci et al. [28] further showed that VTC, behavior, and deep neural networks do not assign the same weights to animal appearance and true animal category. Similarly, in the present study, HOG, Gabor, and ResNet50 convolutional-layer features captured only a subset of computable image properties. Although these features provided a useful way to assess whether measured image structure could account for the neural CI effect, they cannot fully characterize global shape configuration, pose variation, part relations, complex curvature structure, or real-world properties and semantic associations that may be conveyed or triggered by two-dimensional images. In natural object categories, visual form, ecological relevance, and semantic category are often strongly correlated. Therefore, the present study cannot establish that the animacy CI advantage is fully independent of visual form, nor can it establish that the effect is purely semantic. The effect may still reflect the joint contribution of unmeasured shape cues, category structure, and ecological or semantic dimensions. Future studies could combine representational similarity analysis, multilayer deep neural network features, semantic embedding models, and larger stimulus sets [55] to jointly model low-level visual features, mid-level shape structure, biological-form cues, and semantic category information.

### 4.3. Relationship to Behavioral Animal Advantages and Task Design

The present study was motivated by behavioral work on rapid animal detection and categorization. Many behavioral studies have shown that animal stimuli often show processing advantages in rapid categorization, visual search, and change-detection tasks [2,8,16]. The present results provide a candidate representational account for these behavioral observations: under limited input, VOTC multivoxel patterns elicited by animate categories corresponded more selectively to same-category patterns under longer viewing. Such cross-duration category selectivity may provide a favorable representational condition for subsequent rapid detection or categorization decisions.

This interpretation should be considered in light of the task design. The present study did not directly measure explicit animate–inanimate categorization behavior. Instead, participants performed a noise-detection task that was unrelated to category judgments: they responded only when a noise image appeared and did not categorize object images as animate or inanimate. This design helped reduce potential contributions of explicit category judgment, decision strategy, and response-selection differences to the multivoxel patterns, thereby allowing the analysis to focus on VOTC pattern differences elicited under a category-irrelevant task. Correspondingly, noise-detection accuracy and reaction time cannot substitute for behavioral measures of animate–inanimate categorization and cannot directly establish a neural–behavioral association between the animacy CI advantage and behavioral animal advantages. Future studies should combine explicit rapid categorization tasks with neural pattern analyses in the same participants to test whether cross-duration CI predicts individual differences in animal detection or categorization performance.

It is also important to note that the noise-detection task reduced the likelihood of strategic category attention, but it did not eliminate the possible contribution of stimulus-driven attention. Animate stimuli may attract spontaneous attention more readily than some inanimate stimuli. Change-detection studies have shown that animate targets, including humans and other animals, are more readily noticed and detected than inanimate targets, and such animal-detection biases may influence subsequent visual representation strength [13,16]. Thus, the present effect may reflect both differences in category representation and the contribution of stimulus-driven attention. Future studies could combine eye tracking, attentional manipulations, or task-relevance manipulations to further test the role of attention in cross-duration CI for animate categories.

### 4.4. CI Measure, Presentation Duration, and Interpretive Boundaries

The CI measure used in the present study was based on correlation-based MVPA and was intended to assess whether multivoxel patterns for a given category showed category-selective correspondence across the two presentation-duration conditions [17,34]. Specifically, CI tested whether the activity pattern elicited by a category under brief presentation correlated more strongly with the same-category pattern under long presentation than with other-category patterns under long presentation. Thus, higher CI does not simply mean that brief- and long-presentation patterns were globally more similar. Rather, it indicates that the brief-presentation pattern corresponded more selectively to the same-category pattern under long presentation. This measure is therefore well matched to the central question of the present study: whether animate categories, under limited input, more readily elicit VOTC spatial patterns that correspond to same-category patterns under longer viewing.

Based on this measure, the 33 ms condition should be understood as a limited-input condition, not as a direct measure of purely feedforward processing. Brief image presentation followed immediately by masking is commonly used to restrict visual input and to examine the time course of object recognition [4,32]. However, backward masking does not necessarily eliminate local recurrent or feedback processing after stimulus offset. In addition, the temporal resolution of fMRI is insufficient to distinguish millisecond-level feedforward, recurrent, attentional, or semantic processing stages [56]. Therefore, the present results should be interpreted as showing higher cross-duration category selectivity for animate categories under limited input, rather than as demonstrating that the advantage occurred at a specific processing stage. Similarly, VOTC responses in the 500 ms condition may include feedforward, recurrent, attentional, and semantic components. This condition should be treated as a longer-viewing category-pattern condition, not as a complete, final, or neutral visual-semantic representation. Accordingly, CI does not measure the absolute completeness of brief representations, nor does it directly measure the speed of representation formation. Future studies could include multiple presentation durations and combine fMRI with EEG or MEG to characterize how the animacy CI advantage unfolds across input duration and processing time.

Finally, the sample size of the ROI-MVPA analyses limits the precision of effect-size estimation. To mitigate this issue, the present study reported confidence intervals, bootstrap and permutation tests, leave-one-subject-out analyses, top-N sensitivity analyses, and results across three ROI-definition strategies. These analyses supported the direction and consistency of the main effect, but the finding should still be replicated in larger samples and with independent stimulus sets.

## 5. Conclusions

In conclusion, the present study showed that, under brief visual input, animate categories exhibited higher cross-duration category information than inanimate categories in the VOTC. This effect was consistent across multiple ROI-definition strategies, after excluding the human-head category, across different top-N voxel thresholds, and in participant-level robustness analyses. Stimulus-level image-feature analyses indicated that within-category visual homogeneity contributed to CI but did not fully account for the animate-category advantage. These findings should be interpreted with caution because natural object images retain visual-semantic covariation that cannot be fully removed by the present feature-based controls. Thus, animate-related information appears to show relatively high cross-duration category selectivity within the distributed representational space of the ventral occipitotemporal cortex, and this advantage may reflect the joint contribution of animacy-related category organization, visual structure, and category-structure factors.

## Figures and Tables

**Table 1:** Comparison of mean category information between animate and inanimate categories.

ROI	Analysis Set	*df*	*t*	*p*	Cohen’s *d*_z_	95% CI
Joint-ROI	Full stimulus set	17	4.871	<0.001	1.148	[0.092, 0.233]
Joint-ROI	Human-head excluded	17	4.022	<0.001	0.948	[0.061, 0.194]
Group-ROI	Full stimulus set	17	5.551	<0.001	1.308	[0.066, 0.146]
Group-ROI	Human-head excluded	17	3.982	<0.001	0.939	[0.037, 0.120]
Anatomical-ROI	Full stimulus set	17	4.295	<0.001	1.012	[0.035, 0.102]
Anatomical-ROI	Human-head excluded	17	3.398	0.003	0.801	[0.020, 0.085]

Note: Positive *t*-values and positive effect sizes indicate a higher mean CI for animate than for inanimate categories. The human-head-excluded analysis compared the remaining three animate subcategories with the four inanimate subcategories.

**Table 2:** Comparisons of within-subcategory visual homogeneity between animate and inanimate subcategories.

Analysis Set	Feature Space	Animate Mean	Inanimate Mean	95% CI	*p*
Full stimulus set	HOG	0.136	0.094	[−0.079, 0.198]	0.823
Full stimulus set	Gabor	0.236	0.197	[−0.156, 0.240]	0.667
Full stimulus set	ResNet50 layer 1	0.290	0.253	[−0.225, 0.276]	0.779
Full stimulus set	ResNet50 layer 2	0.274	0.277	[−0.232, 0.211]	0.973
Full stimulus set	ResNet50 layer 3	0.303	0.253	[−0.150, 0.287]	0.718
Human-head excluded	HOG	0.060	0.094	[−0.100, 0.031]	0.425
Human-head excluded	Gabor	0.151	0.197	[−0.196, 0.087]	0.795
Human-head excluded	ResNet50 layer 1	0.209	0.253	[−0.285, 0.141]	0.857
Human-head excluded	ResNet50 layer 2	0.205	0.277	[−0.275, 0.108]	0.712
Human-head excluded	ResNet50 layer 3	0.194	0.253	[−0.184, 0.059]	0.630

Note: Confidence intervals were estimated using bootstrap resampling. The *p* values were obtained from two-tailed permutation tests using subcategory labels as the unit of permutation. The full stimulus set included four animate and four inanimate subcategories. The human-head-excluded analysis included three animate and four inanimate subcategories.

**Table 3:** Effects of animacy and Visual homogeneity PC1 on category information in baseline and visual-control models.

Model	ROI	Analysis Set	Predictor	*β*	*SE*	*t*	*p*	95% CI
Baseline	Joint-ROI	Full stimulus set	Animacy	0.163	0.036	4.55	<0.001	[0.093, 0.233]
	Joint-ROI	Human-head excluded	Animacy	0.127	0.034	3.72	<0.001	[0.060, 0.195]
	Group-ROI	Full stimulus set	Animacy	0.106	0.020	5.19	<0.001	[0.066, 0.146]
	Group-ROI	Human-head excluded	Animacy	0.080	0.022	3.68	<0.001	[0.037, 0.122]
	Anatomical-ROI	Full stimulus set	Animacy	0.068	0.017	4.02	<0.001	[0.035, 0.102]
	Anatomical-ROI	Human-head excluded	Animacy	0.053	0.017	3.14	0.002	[0.020, 0.086]
Visual-control	Joint-ROI	Full stimulus set	Animacy	0.154	0.035	4.36	<0.001	[0.085, 0.224]
	Joint-ROI	Full stimulus set	Visual homogeneity PC1	0.035	0.010	3.53	<0.001	[0.016, 0.054]
	Joint-ROI	Human-head excluded	Animacy	0.130	0.035	3.73	<0.001	[0.061, 0.198]
	Joint-ROI	Human-head excluded	Visual homogeneity PC1	0.006	0.016	0.38	0.700	[−0.025, 0.038]
	Group-ROI	Full stimulus set	Animacy	0.100	0.020	4.86	<0.001	[0.059, 0.140]
	Group-ROI	Full stimulus set	Visual homogeneity PC1	0.026	0.008	3.31	<0.001	[0.011, 0.042]
	Group-ROI	Human-head excluded	Animacy	0.082	0.022	3.65	<0.001	[0.038, 0.125]
	Group-ROI	Human-head excluded	Visual homogeneity PC1	0.006	0.011	0.48	0.629	[−0.017, 0.028]
	Anatomical-ROI	Full stimulus set	Animacy	0.064	0.017	3.84	<0.001	[0.031, 0.097]
	Anatomical-ROI	Full stimulus set	Visual homogeneity PC1	0.016	0.006	2.82	0.005	[0.005, 0.028]
	Anatomical-ROI	Human-head excluded	Animacy	0.054	0.018	3.09	0.002	[0.020, 0.089]
	Anatomical-ROI	Human-head excluded	Visual homogeneity PC1	0.005	0.009	0.56	0.575	[−0.012, 0.022]

Note. Animacy was effect-coded as animate = 0.5 and inanimate = −0.5; therefore, the animacy coefficient represents the mean CI difference between animate and inanimate categories. Visual homogeneity PC1 refers to the composite within-subcategory visual homogeneity index derived from HOG, Gabor, and ResNet50 layers 1–3 feature spaces. Standard errors are participant-clustered robust standard errors.

## Data Availability

The data presented in this study are available on request from the corresponding author due to privacy or ethical restrictions. Supporting materials have been deposited on the Open Science Framework (OSF) and can be accessed at https://osf.io/pfz5b/overview?view_only=71fd35128cca4164b892365b1c1a5ccb (accessed on 24 June 2026).

## Supplementary Materials

The following supporting information can be downloaded at https://www.mdpi.com/article/10.3390/brainsci16070668/s1. Supplementary Methods S1: Image-feature extraction methods; Supplementary Tables S1–S3: Whole-brain activation analyses; Supplementary Table S4: Post hoc pairwise comparisons of category information; Supplementary Table S5: Visual homogeneity PC1 control-model results; Supplementary Tables S6–S8: Subcategory-level CI summaries and animate-subcategory sensitivity analyses; Supplementary Tables S9 and S10: Top-N voxel-selection sensitivity analyses and leave-one-subject-out stability analyses; Supplementary Tables S11–S15: Associations between scanner-task behavioral performance and neural CI; Supplementary Tables S16–S18: Visual feature extraction, visual homogeneity PCA, and PC1 scores; Supplementary Figures S1–S6: Group-ROI, Anatomical-ROI, top-N sensitivity, leave-one-subject-out stability, and visual-feature similarity matrices.
