# Supplementary material for: Animate Categories Show Higher Cross-Duration Representational Selectivity in Ventral Occipitotemporal Cortex Under Brief Visual Input
Source: Brain Sci. 2026 Jun 26;16(7):668. doi: 10.3390/brainsci16070668 (PMC13407226; doi:10.3390/brainsci16070668)
Supplement: Supplementary file 1 [file brainsci-16-00668-s001.zip › Supplementary Materials-Figures S1–S6.pdf]

Supplementary Materials

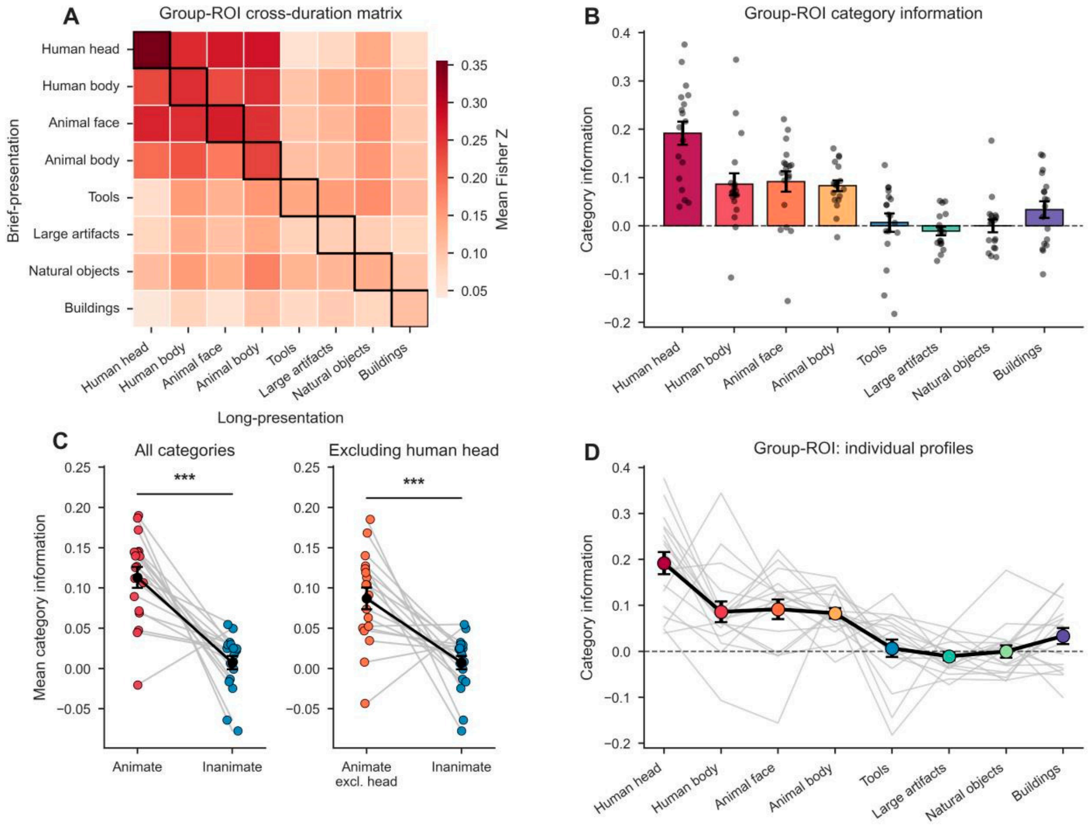

Supplementary Figure S1. Cross-duration category information in the Group-ROI.

A. Cross-duration correlation matrix between the brief- and long-presentation conditions in the Group-ROI. Rows and columns indicate category-specific activity patterns in the brief- and long-presentation conditions, respectively. Each cell represents the mean Pearson correlation coefficient after Fisher z-transformation. The black squares indicate within-category correlations across the two presentation conditions. B. Category information for the eight object subcategories in the Group-ROI. The gray circles represent the individual participants, the bars represent the group means, and the error bars represent the standard errors of the mean. C. Comparison of category information between animate and inanimate categories. The left panel shows the comparison including all animate and inanimate subcategories, and the right panel shows the comparison after excluding the human-head category. The gray lines indicate paired data from the

same participant; the black circles and error bars indicate the group means and standard errors of the mean. D. Individual category-information profiles in the Group-ROI. The gray lines indicate category information across the eight object subcategories for each participant, the black line indicates the group mean, and the error bars indicate the standard errors of the mean. The dashed line indicates zero category information. *** $p < .001$.

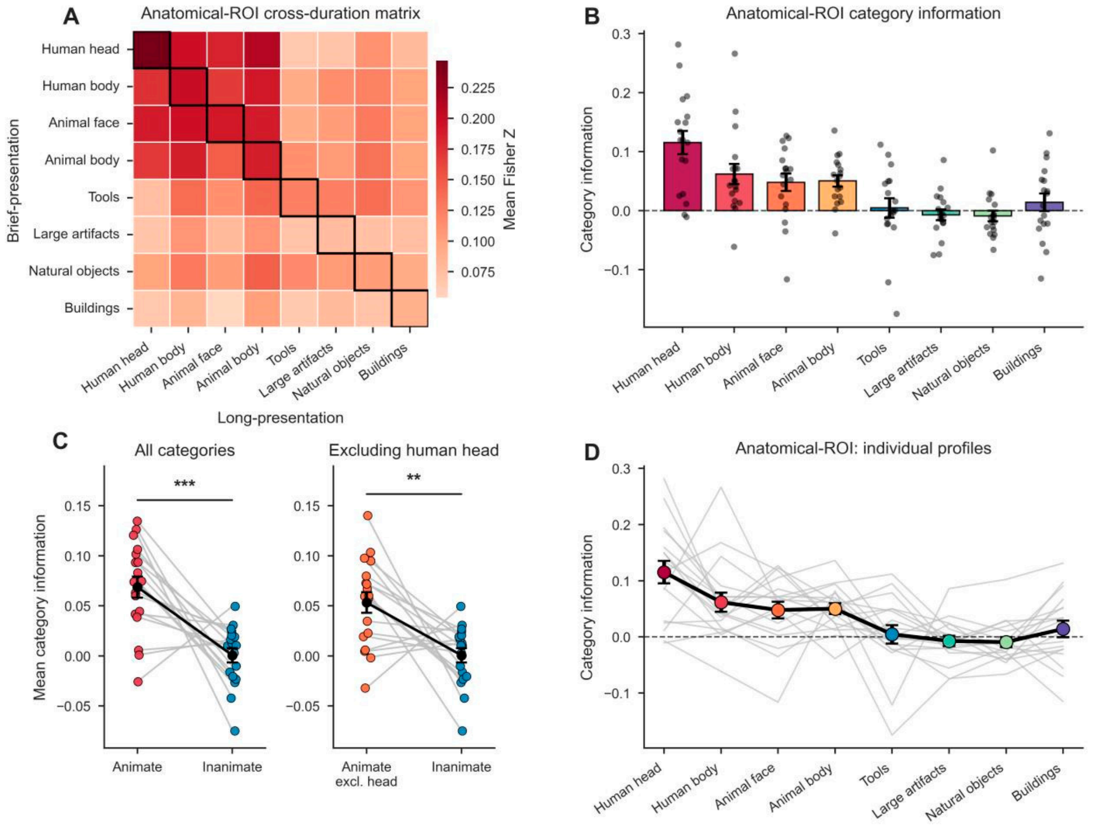

Supplementary Figure S2. Cross-duration category information in the Anatomical-ROI.

A. Cross-duration correlation matrix between the brief- and long-presentation conditions in the Anatomical-ROI. Rows and columns indicate category-specific activity patterns in the brief- and long-presentation conditions, respectively. Each cell represents the mean Pearson correlation coefficient after Fisher *z*-transformation. The black squares indicate within-category correlations across the two presentation conditions. B. Category information for the eight object subcategories in the Anatomical-ROI. The gray circles represent the individual participants, the bars indicate the group means, and the error bars indicate the standard errors of the mean. C. Comparison of

category information between animate and inanimate categories. The left panel shows the comparison including all animate and inanimate subcategories, and the right panel shows the comparison after excluding the human-head category. The gray lines indicate paired data from the same participant; the black circles and error bars indicate the group means and standard errors of the mean. D. Individual category-information profiles in the Anatomical-ROI. The gray lines indicate category information across the eight object subcategories for each participant, the black line indicates the group mean, and the error bars indicate the standard errors of the mean. The dashed line indicates zero category information. *** $p < .001$.

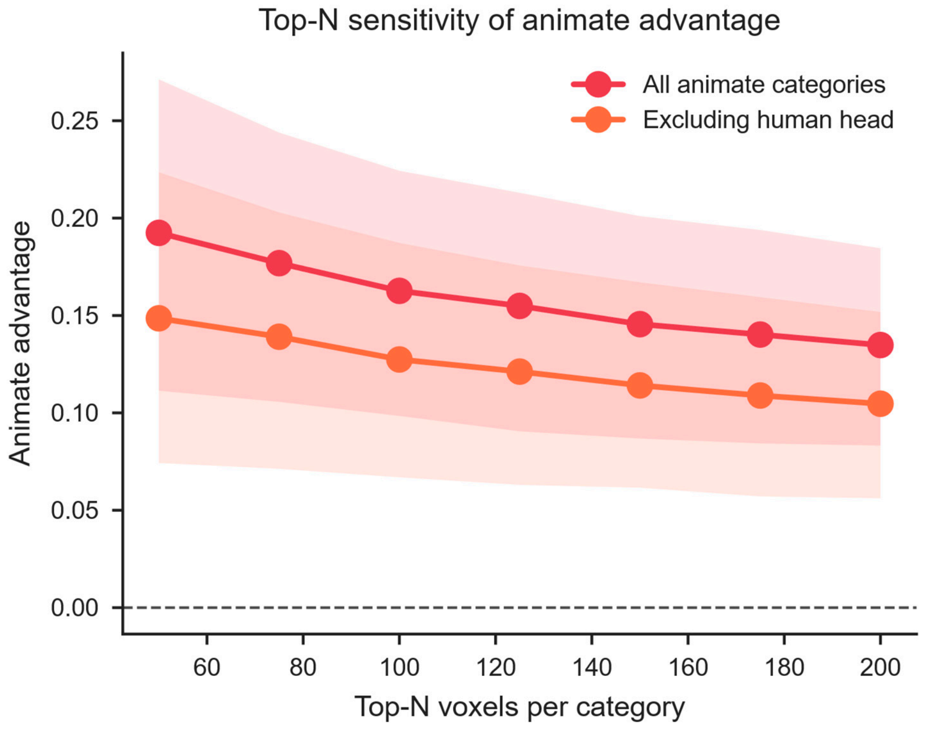

Supplementary Figure S3. Animate-category advantage across different top-N voxel selections. This figure shows the animate-category advantage in the Joint-ROI across different top-N voxel selections. The x-axis indicates the number of top-ranked voxels selected for each category when defining the Joint-ROI, and the y-axis indicates the category-information difference between animate and inanimate categories. The solid line shows the animate advantage when all animate categories were included, whereas the dashed line shows the animate advantage after excluding the human-head category. Error bars indicate standard errors of the mean. Across the top 50 to top

200 voxels, the animate categories consistently showed higher category information than the inanimate categories, indicating that the main effect did not depend on a single voxel-selection threshold.

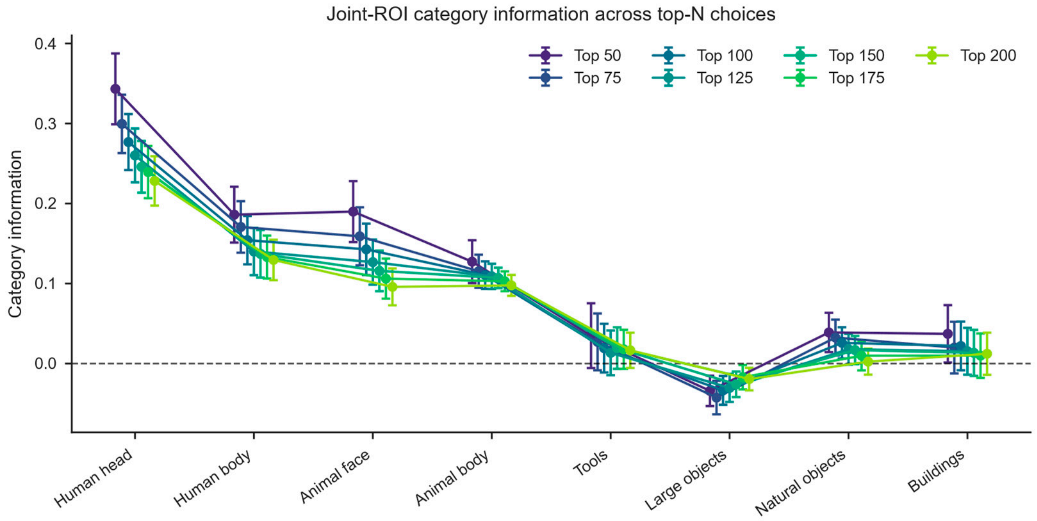

Supplementary Figure S4. Category information across different top-N voxel selections.

This figure shows category information for the eight object subcategories in the Joint-ROI across different top-N voxel selections. The x-axis indicates object subcategories, and the y-axis indicates category information. Different colored lines indicate different top-N voxel-selection thresholds. The overall category-information profile was similar across top-N settings, with animate categories, including human head, human body, animal face, and animal body, generally showing higher category information than inanimate categories. This pattern indicates that the category-information profile was stable across different voxel-selection thresholds.

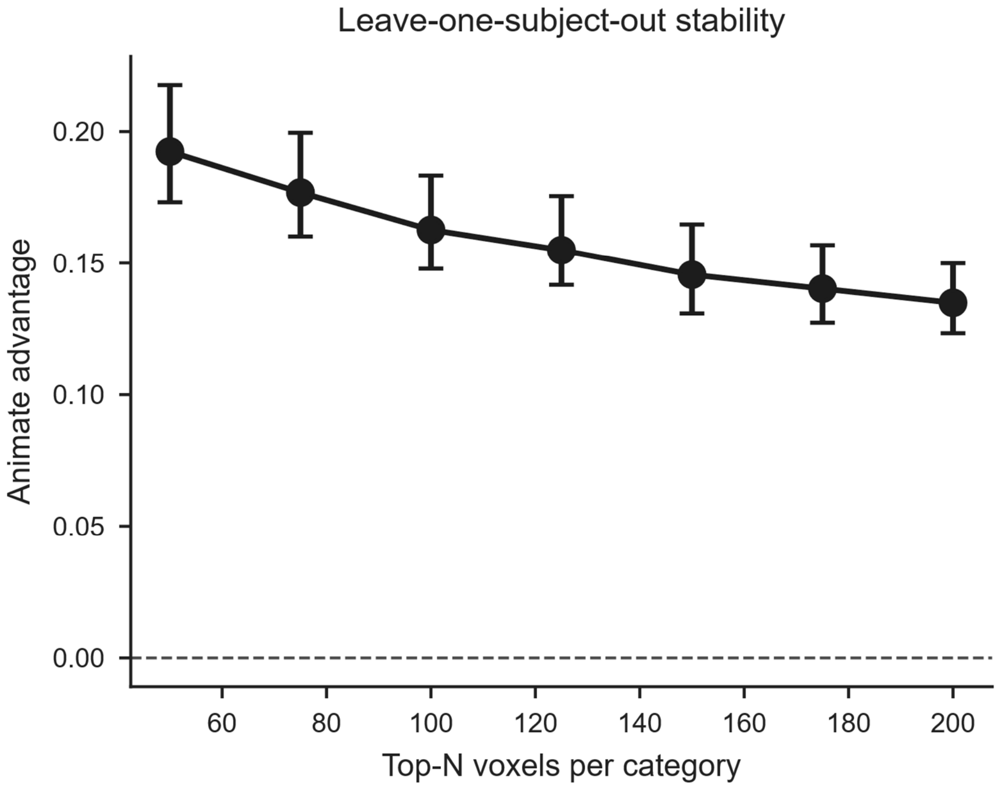

Supplementary Figure S5. Leave-one-subject-out stability across different top-N voxel selections. This figure shows the leave-one-subject-out stability of the animate-category advantage in the Joint-ROI across different top-N voxel selections. The x-axis indicates the number of top-ranked voxels selected for each category when defining the Joint-ROI, and the y-axis indicates the animate-category advantage recalculated after excluding one participant at a time. Each point represents one leave-one-subject-out iteration, and the line shows the mean trend across the top-N settings. The animate-category advantage remained positive across all top-N settings and all leave-one-subject-out iterations, indicating that the effect was not driven by any single participant and was stable across voxel-selection thresholds.

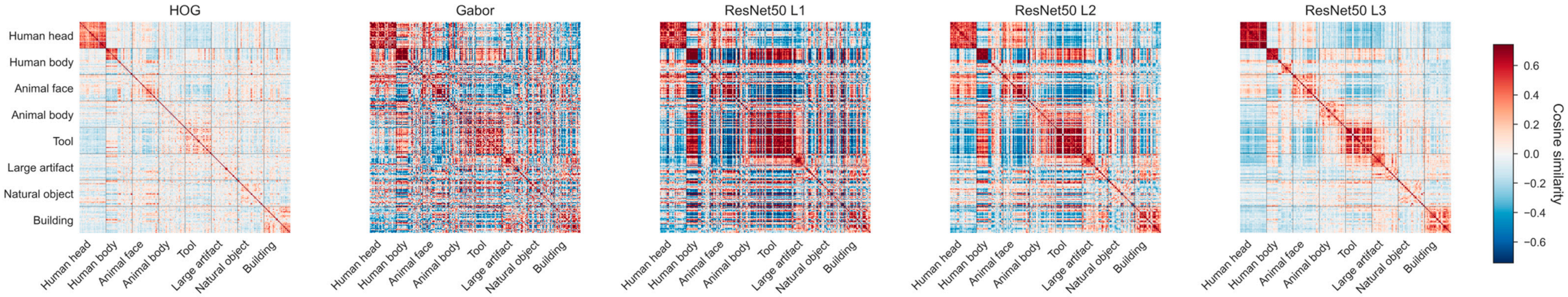

Supplementary Figure S6. Pairwise cosine similarity matrices of stimulus images in the HOG, Gabor, ResNet50 layers 1 − 3 space.

Each matrix (size 256 × 256) represents the pairwise cosine similarity between all stimulus images. Images are grouped into eight subcategories: human head, human body, animal face, animal body, tool, large artifact, natural object, and building. Red indicates high positive similarity; blue indicates low or negative similarity. Diagonal elements (self-similarity = 1) appear in red. The ordering of images follows the subcategory sequence listed above. This figure illustrates the overall similarity structure of the stimulus set in each feature space.
