# Supplementary material for: Animate Categories Show Higher Cross-Duration Representational Selectivity in Ventral Occipitotemporal Cortex Under Brief Visual Input
Source: Brain Sci. 2026 Jun 26;16(7):668. doi: 10.3390/brainsci16070668 (PMC13407226; doi:10.3390/brainsci16070668)
Supplement: Supplementary file 1 [file brainsci-16-00668-s001.zip › Supplementary Materials-Methods S1-Image Feature Extraction.pdf]

Supplementary Methods: Image-Feature Extraction

We extracted three types of image-computable features to quantify the visual similarity structure of the stimulus images: histograms of oriented gradients (HOG), Gabor filter features, and intermediate convolutional features from a ResNet50 model pretrained on ImageNet.

1. HOG Features

HOG features were used to characterize local edge orientations, local shape information, and contour structure. All images were converted to grayscale and resized to 128 × 128 pixels before feature extraction. HOG features were extracted using 9 orientation bins, a cell size of 16 × 16 pixels, a block size of 2 × 2 cells, and L2-Hys block normalization. This procedure yielded a 1,764-dimensional feature vector for each image.

2. Gabor Filter Features

Gabor filter features were used to characterize orientation energy, spatial-frequency structure, and coarse texture statistics. All images were converted to grayscale and resized to 128 × 128 pixels. A Gabor filter bank was constructed using four spatial frequencies (0.05, 0.10, 0.20, and 0.30) and four orientations (0°, 45°, 90°, and 135°). For the magnitude distribution of each filter response, five summary statistics were extracted: the mean, standard deviation, 25th percentile, median, and 75th percentile. This procedure yielded an 80-dimensional feature vector for each image (4 frequencies × 4 orientations × 5 statistics).

3. ResNet50 Intermediate-Layer Features

Intermediate convolutional features were extracted from a ResNet50 model pretrained on ImageNet. These features were treated as image-computable convolutional feature spaces that capture increasingly complex visual structure, rather than as direct neural measurements or purely semantic representations. All grayscale

images were converted to three-channel input by replicating the single grayscale channel, resized to 224 × 224 pixels, and normalized using the ImageNet mean values (0.485, 0.456, and 0.406) and standard deviations (0.229, 0.224, and 0.225). Images were passed through the initial layers of the model, including conv1, bn1, relu, and maxpool. The outputs of layer1, layer2, and layer3 were then extracted separately. Each layer output was reduced to a one-dimensional vector using adaptive average pooling. This procedure yielded three feature vectors for each image: 256 dimensions for layer1, 512 dimensions for layer2, and 1,024 dimensions for layer3.
