# Supplementary material for: Animate Categories Show Higher Cross-Duration Representational Selectivity in Ventral Occipitotemporal Cortex Under Brief Visual Input
Source: Brain Sci. 2026 Jun 26;16(7):668. doi: 10.3390/brainsci16070668 (PMC13407226; doi:10.3390/brainsci16070668)
Supplement: Supplementary file 1 [file brainsci-16-00668-s001.zip › Supplementary Tables S11–S18- Behavior-Neural Correlations and Visual Feature Analyses.pdf]

Supplementary Materials: Behavior–Neural Correlation Analyses

We analyzed behavioral data from the fMRI scanner task, that is, the noise-detection task, to determine whether the neural category-information effects were associated with task performance. At the participant level, scanner-task behavioral measures were correlated with the category information (CI) animacy advantage (animate CI minus inanimate CI) separately for the brief- and long-presentation conditions, and separately for the Joint-ROI, Group-ROI, and Anatomical-ROI. Because the sample size was modest and behavioral accuracy was close to the ceiling in some conditions, Spearman correlations were used as the primary tests. At the category level, each participant's behavioral accuracy across the eight stimulus categories was correlated with the corresponding eight category-wise CI values. Finally, category-level regression models tested whether animacy still predicted CI after controlling for category-wise behavioral accuracy.

At the participant level, scanner-task performance was not reliably associated with the neural animacy advantage in any ROI. At the exploratory category level, behavioral accuracy and neural CI showed a positive correspondence in the brief-presentation condition but not in the long-presentation condition, where behavioral accuracy was near the ceiling. Importantly, category-level control models showed that animacy remained a significant predictor of CI after controlling for category-wise scanner-task accuracy, whereas behavioral accuracy itself was not a significant predictor after FDR correction.

Table S11. Scanner-task behavioral performance.

Duration	Behavioral measure	Mean	*SD*	95% CI
Brief	Overall ACC	0.933	0.034	[0.916, 0.950]
Brief	Noise ACC	0.830	0.118	[0.771, 0.889]
Brief	Noise-hit RT mean	644.062	125.212	[581.796, 706.329]
Long	Overall ACC	0.993	0.011	[0.988, 0.999]
Long	Noise ACC	0.983	0.053	[0.956, 1.009]
Long	Noise-hit RT mean	579.994	143.888	[508.441, 651.548]

Note: Overall ACC = overall task accuracy across all trials; Noise ACC = accuracy for noise-target trials only; Noise-hit RT mean = mean response time, in milliseconds, for correctly detected noise-target trials.

Table S12. Comparison of behavioral performance between brief- and long-presentation conditions.

Behavioral measure	Brief	Long	Difference	*t* test	FDR *p*
Overall ACC	0.933	0.993	0.060	t(17) = 7.13	< .001
Noise ACC	0.830	0.983	0.153	t(17) = 4.83	< .001
Noise-hit RT mean	644.062	579.994	-64.068	t(17) = -1.52	0.147
Noise-hit RT median	612.000	530.028	-81.972	t(17) = -2.12	0.058

Note: Difference = Long - Brief. Response times are reported in milliseconds. p values are FDR-corrected across behavioral measures.

Table S13. Participant-level correlations between scanner-task behavioral performance and neural animacy advantage.

Duration	ROI	Behavioral Measure	Spearman rho	Uncorrected *p*	FDR *p*
Brief	Anatomical-ROI	Overall ACC	0.13	0.611	0.814

Brief	Anatomical-ROI	Noise ACC	-0.14	0.584	0.814
Brief	Anatomical-ROI	Noise-hit RT mean	0.17	0.499	0.807
Brief	Group-ROI	Overall ACC	0.14	0.582	0.814
Brief	Group-ROI	Noise ACC	-0.21	0.398	0.801
Brief	Group-ROI	Noise-hit RT mean	0.11	0.651	0.854
Brief	Joint-ROI	Overall ACC	0.29	0.247	0.714
Brief	Joint-ROI	Noise ACC	0.05	0.847	0.961
Brief	Joint-ROI	Noise-hit RT mean	-0.03	0.919	0.961
Long	Anatomical-ROI	Overall ACC	-0.05	0.850	0.991
Long	Anatomical-ROI	Noise ACC	0.09	0.711	0.991
Long	Anatomical-ROI	Noise-hit RT mean	-0.04	0.861	0.991
Long	Group-ROI	Overall ACC	0.05	0.833	0.991
Long	Group-ROI	Noise ACC	0.20	0.437	0.991
Long	Group-ROI	Noise-hit RT mean	0.14	0.570	0.991
Long	Joint-ROI	Overall ACC	0.11	0.656	0.991
Long	Joint-ROI	Noise ACC	0.19	0.445	0.991
Long	Joint-ROI	Noise-hit RT mean	-0.18	0.473	0.991

Note: FDR correction was performed within each presentation condition.

Table S14. Exploratory category-level behavior–CI correspondence.

Duration	ROI	*n*	Mean rho	*t* test	*p*	95% CI rho
Brief	Anatomical-ROI	18	0.36	$t(17) = 4.76$	< .001	[0.22, 0.53]
Brief	Group-ROI	18	0.41	$t(17) = 5.58$	< .001	[0.28, 0.57]
Brief	Joint-ROI	18	0.44	$t(17) = 5.25$	< .001	[0.31, 0.63]
Long	Anatomical-ROI	8	-0.06	$t(7) = -0.38$	0.712	[-0.44, 0.33]
Long	Group-ROI	8	-0.08	$t(7) = -0.51$	0.712	[-0.45, 0.30]
Long	Joint-ROI	8	-0.13	$t(7) = -0.93$	0.573	[-0.45, 0.21]

Note: For each participant, Spearman correlations were computed across the eight categories and then tested at the group level after Fisher Z-transformation.

Table S15. Category-level behavioral accuracy control regression.

Duration	ROI	Predictor	*β*	*SE*	*t*	*p*	95% CI
Brief	Anatomical-ROI	Animacy	0.067	0.018	3.68	0.003	[0.028, 0.105]
Brief	Anatomical-ROI	Category behavioral accuracy	0.002	0.004	0.43	0.676	[-0.007, 0.011]
Brief	Group-ROI	Animacy	0.098	0.023	4.34	0.001	[0.051, 0.146]
Brief	Group-ROI	Category behavioral accuracy	0.009	0.006	1.51	0.169	[-0.004, 0.022]
Brief	Joint-ROI	Animacy	0.147	0.036	4.11	0.002	[0.071, 0.222]
Brief	Joint-ROI	Category behavioral accuracy	0.020	0.010	2.09	0.067	[-0.000, 0.040]
Long	Anatomical-ROI	Animacy	0.068	0.017	3.99	0.001	[0.032, 0.105]
Long	Anatomical-ROI	Category behavioral accuracy	-0.004	0.006	-0.64	0.593	[-0.015, 0.008]
Long	Group-ROI	Animacy	0.106	0.021	5.16	< .001	[0.063, 0.149]

Long	Group-ROI	Category behavioral accuracy	-0.004	0.007	-0.54	0.593	[-0.019, 0.011]
Long	Joint-ROI	Animacy	0.163	0.036	4.53	< .001	[0.087, 0.239]
Long	Joint-ROI	Category behavioral accuracy	-0.007	0.011	-0.67	0.593	[-0.030, 0.015]

Note: Model: CI ~ animacy + category behavioral accuracy + subject fixed effects. Behavioral accuracy was standardized within each duration condition. Cluster-robust standard errors were clustered by subject. p values are FDR-corrected within each duration condition.

Table S16. Within-category visual similarity for each stimulus subcategory across five image-feature spaces.

	HOG	Gabor	ResNet50 layer1	ResNet50 layer2	ResNet50 layer3
human head	0.364	0.490	0.533	0.481	0.629
human body	0.064	0.172	0.158	0.134	0.165
animal face	0.103	0.215	0.280	0.285	0.219
animal body	0.014	0.066	0.188	0.195	0.199
tool	0.153	0.399	0.597	0.571	0.433
large artifact	0.035	0.157	0.185	0.185	0.205
natural object	0.049	0.042	0.020	0.064	0.097
building	0.141	0.190	0.212	0.288	0.278

Note: Values represent the mean pairwise cosine similarity among all images within each subcategory. HOG = histogram of oriented gradients.

Table S17. PCA of visual-homogeneity indices.

Table S17A. Explained variance ratios from PCA of visual homogeneity indices.

	Explained Variance Ratio	Cumulative explained variance ratio
PC1	0.910	0.910
PC2	0.074	0.984
PC3	0.010	0.993
PC4	0.006	0.999
PC5	0.001	1.000

Note: PCA was performed of the five within-category visual-similarity indices (HOG, Gabor, ResNet50 layer1–3) across the eight subcategories. Each index was standardized prior to PCA.

Table S17B. Principal component loadings from PCA for visual homogeneity indices.

feature	PC1	PC2	PC3	PC4	PC5
HOG	0.421	-0.711	0.053	0.452	-0.332
Gabor	0.461	-0.044	-0.780	-0.110	0.405
ResNet50 layer1	0.449	0.456	-0.106	-0.224	-0.727
ResNet50 layer2	0.444	0.486	0.339	0.571	0.354
ResNet50 layer3	0.459	-0.220	0.512	-0.638	0.267

Note: Loadings indicate the direction and magnitude of the contribution of each visual homogeneity index to the corresponding principal component. HOG = histogram of oriented gradients.

Table S18. Visual PCA scores for each stimulus subcategory.

	Visual PC1	Visual PC2	Visual PC3	Visual PC4	Visual PC5
Human head	4.078	-0.980	0.044	-0.119	-0.027
Human body	-1.336	-0.193	-0.375	-0.099	-0.010

Animal face	-0.175	0.214	-0.173	0.203	-0.077
Animal body	-1.537	0.392	0.386	-0.192	-0.071
Tool	2.785	1.174	-0.042	0.058	0.005
Large artifact	-1.185	0.177	-0.093	-0.226	0.111
Natural object	-2.535	-0.510	0.035	0.124	-0.041
Building	-0.095	-0.274	0.217	0.251	0.110

Note: Visual PC1 was used as the composite visual homogeneity index in the regression analyses reported in the main text. PCA was computed based on the five within-category visual-similarity indices.
