# Supplementary material for: Animate Categories Show Higher Cross-Duration Representational Selectivity in Ventral Occipitotemporal Cortex Under Brief Visual Input
Source: Brain Sci. 2026 Jun 26;16(7):668. doi: 10.3390/brainsci16070668 (PMC13407226; doi:10.3390/brainsci16070668)
Supplement: Supplementary file 1 [file brainsci-16-00668-s001.zip › Supplementary Tables S1–S5- Whole-Brain Activation, Pairwise CI Comparisons, and Visual-Control Models.pdf]

Supplementary Materials: Whole-Brain Activation Analyses

These tables provide detailed whole-brain activation results from the univariate analyses. Table S1 presents the direct contrasts between animate and inanimate categories separately for the brief- and long-presentation conditions. Table S2 presents the activation results for each object subcategory relative to the noise baseline under both presentation conditions. Table S3 presents the direct presentation comparisons of subcategory-level activation.

Table S1. Whole-brain activation differences between animate and inanimate categories.

Condition	Contrast	Hemisphere	Area	Peak MNI coordinates			Cluster size (voxels)	Cluster volume (mm³)	*t*
				x	y	z			
Brief-presentation	animate > inanimate	R	Inferior occipital gyrus	42	-78	-3	317	8559	11.088
		R	Middle temporal gyrus						
		R	Middle occipital gyrus						
		R	Fusiform gyrus						
		R	Superior temporal gyrus						
		L	Middle occipital gyrus	-39	-72	-12	70	1890	6.438
		L	Inferior occipital gyrus						
		L	Fusiform gyrus						
	inanimate > animate	R	Fusiform gyrus	27	-42	-21	37	999	-6.081
		R	Parahippocampal gyrus						
		L	Inferior frontal gyrus	-30	24	3	59	1593	-6.173
		R	Inferior frontal gyrus	39	21	6	33	891	-6.624
		L	Superior frontal gyrus	-3	15	51	62	1674	-6.774
		L	Medial frontal gyrus						
Long-presentation	animate > inanimate	R	Fusiform gyrus	42	-54	-15	58	1566	7.028
		L	Inferior occipital gyrus	-42	-75	-12	43	1161	6.358
		L	Middle occipital gyrus						
		L	Fusiform gyrus						

		R	Middle temporal gyrus	39	-81	-9	278	7506	6.924
		R	Middle occipital gyrus						
		R	Superior temporal gyrus						
		R	Inferior occipital gyrus						
		L	Middle temporal gyrus	-48	-66	12	50	1350	5.757
		L	Superior temporal gyrus						
	inanimate > animate	L	Parahippocampal gyrus	-27	-42	-12	79	2133	-9.036
		L	Fusiform gyrus						
		R	Fusiform gyrus	30	-42	-12	98	2646	-7.459

Table S2. Whole-brain activations for each subcategory relative to noise.

Condition	Contrast	Hemisphere	Area	Peak MNI coordinates			Cluster size	Cluster volume	*t*
				x	y	z	(voxels)	(mm^3)	
Brief-presentation	Human head > noise	R	Middle occipital gyrus	45	-75	-6	130	3510	9.701
			Fusiform gyrus						
			Inferior temporal gyrus						
			Inferior occipital gyrus						
	Human body > noise	R	Middle temporal gyrus	45	-75	3	370	9990	9.612
			Middle temporal gyrus						
			Inferior temporal gyrus						
			Middle occipital gyrus						
			Fusiform gyrus						
			Superior temporal gyrus						
		L	Inferior frontal gyrus	-39	33	-15	30	810	5.222

	L	Middle occipital gyrus	-45	-75	-3	150	4050	8.342
		Inferior occipital gyrus						
		Middle temporal gyrus						
Animal head > noise	L	Middle occipital gyrus	-45	-78	-3	160	4320	7.953
		Inferior occipital gyrus						
		Fusiform gyrus						
	R	Middle occipital gyrus	45	-72	-6	259	6993	10.000
		Inferior temporal gyrus						
		Inferior occipital gyrus						
		Fusiform gyrus						
Animal body > noise	L	Parahippocampal gyrus	-30	-30	-18	39	1053	6.684
		Fusiform gyrus						
	L	Parahippocampal gyrus	-30	-12	-21	30	810	7.297
	R	Medial frontal gyrus	0	30	-15	92	2484	5.688
		Medial orbital frontal gyrus						
		Medial orbital frontal gyrus						
	R	Middle occipital gyrus	45	-75	3	160	4320	6.846
		Middle temporal gyrus						
		Inferior temporal gyrus						
	L	Middle occipital gyrus	-45	-78	-3	42	1134	8.608
	L	Precuneus	-12	-54	21	35	945	6.455
Tool > noise	L	Fusiform gyrus	-27	-30	-15	84	2268	8.599
		Parahippocampal gyrus						
	R	Parahippocampal gyrus	21	-3	-21	34	918	7.801
	R	Parahippocampal gyrus	24	-33	-12	41	1107	5.846

			Fusiform gyrus						
		R	Medial frontal gyrus	6	45	-15	53	1431	6.295
		R	Inferior temporal gyrus	45	-75	3	159	4293	7.361
			Middle temporal gyrus						
			Middle occipital gyrus						
		L	Middle occipital gyrus	-45	-75	0	130	3510	6.904
			Inferior occipital gyrus						
		L	Precuneus	-6	-51	12	47	1269	6.135
			Posterior cingulate gyrus						
	Natural objects > noise	L	Orbital part of inferior frontal gyrus	-45	33	-9	46	1242	6.388
		R	Medial frontal gyrus	6	39	-12	90	2430	6.474
			Anterior cingulate gyrus						
			Limbic lobe						
	Building > noise	L	Parahippocampal gyrus	-33	-45	-6	33	891	6.149
	Large artifacts > noise		no cluster exceeded the reporting threshold.						
Long-presentation	Human head > noise	L	Superior temporal gyrus	-42	12	-30	34	918	6.160
			Middle temporal pole						
		L	Fusiform gyrus	-39	-48	-21	36	972	6.290
		R	Inferior occipital gyrus	39	-48	-18	255	6885	8.656
			Middle occipital gyrus						
			Fusiform gyrus						
			Inferior occipital gyrus						
		L	Middle occipital gyrus	-42	-81	-6	110	2970	9.232
			Inferior occipital gyrus						

	L	Inferior frontal gyrus	-27	27	-18	75	2025	8.035
	L	Limbic lobe	-6	-57	18	31	837	5.709
		Posterior cingulate gyrus						
		Precuneus						
	L	Middle temporal gyrus	-42	-60	24	70	1890	7.231
Humanbody > noise	R	Middle occipital gyrus	45	-78	0	258	6966	8.343
		Inferior temporal gyrus						
		Inferior occipital gyrus						
		Fusiform gyrus						
		Inferior temporal gyrus						
	L	Fusiform gyrus	-33	-33	-21	54	1458	7.653
	L	Middle occipital gyrus	-42	-78	-6	120	3240	8.501
		Inferior occipital gyrus						
Animalhead > noise	L	Inferior occipital gyrus	-42	-81	-3	248	6696	6.501
		Fusiform gyrus						
		Middle occipital gyrus						
		Inferior occipital gyrus						
	R	Middle occipital gyrus	39	-48	-15	386	10422	7.749
		Inferior temporal gyrus						
		Fusiform gyrus						
		Middle occipital gyrus						
		Inferior occipital gyrus						
	L	Fusiform gyrus	-36	-36	-21	40	1080	6.285
	L	Inferior occipital gyrus	-42	-81	-3	126	3402	9.153
		Middle occipital gyrus						

		Fusiform gyrus						
	R	Middle occipital gyrus	45	-75	-3	295	7965	9.892
		Inferior occipital gyrus						
		Inferior temporal gyrus						
		Middle temporal gyrus						
		Fusiform gyrus						
		Posterior cingulate gyrus	0	-51	9	47	1269	6.042
		Limbic lobe						
	L	Middle occipital gyrus	-42	-75	33	30	810	4.835
Animalbody > noise	L	Fusiform gyrus	-36	-36	-21	40	1080	6.285
	L	Inferior occipital gyrus	-42	-81	-3	126	3402	9.153
		Middle occipital gyrus						
		Fusiform gyrus						
	R	Middle occipital gyrus	45	-75	-3	295	7965	9.892
		Inferior occipital gyrus						
		Inferior temporal gyrus						
		Middle temporal gyrus						
		Fusiform gyrus						
		Posterior cingulate gyrus	0	-51	9	47	1269	6.042
		Limbic lobe						
	L	Middle occipital gyrus	-42	-75	33	30	810	4.835
Tool > noise	L	Parahippocampal gyrus	-30	-30	-15	93	2511	6.933
		Fusiform gyrus						
		Parahippocampal gyrus						
	L	Angular gyrus	-51	-72	30	45	1215	6.124

Large artifacts > noise	L	Fusiform gyrus	-36	-39	-18	153	4131	7.949
		Parahippocampal gyrus						
	R	Middle occipital gyrus	36	-81	18	290	7830	7.687
		Fusiform gyrus						
		Parahippocampal gyrus						
		Middle temporal gyrus						
	L	Inferior occipital gyrus	-42	-75	-6	39	1053	7.121
	L	Middle occipital gyrus	-33	-81	27	149	4023	7.962
		Superior occipital gyrus						
Natural objects > noise	L	Fusiform gyrus	-36	-60	-12	233	6291	8.126
		Inferior occipital gyrus						
		Parahippocampal gyrus						
		Middle occipital gyrus						
	R	Inferior temporal gyrus	45	-78	0	185	4995	9.449
		Fusiform gyrus						
		Middle occipital gyrus						
		Parahippocampal gyrus						
	R	Middle occipital gyrus	33	-81	15	54	1458	8.412
	L	Middle occipital gyrus	-30	-90	12	82	2214	7.251
Building > noise	L	Fusiform gyrus	-33	-36	-18	107	2889	7.804
		Parahippocampal gyrus						
	R	Fusiform gyrus	33	-36	-15	114	3078	6.924
		Parahippocampal gyrus						
	R	Middle occipital gyrus	33	-81	9	316	8532	7.165
		Middle temporal gyrus						

			Middle occipital gyrus						
		L	Middle occipital gyrus	-39	-81	36	176	4752	7.102
			Middle temporal gyrus						

Table S3. Direct duration comparisons for subcategory-level activation

Condition	Contrast	Hemisphere	Area	Peak MNI coordinates			Cluster size (voxels)	Cluster volume (mm^3)	*t*
				x	y	z			
Long > Brief	Large artifacts > noise	R	Middle occipital gyrus	36	-84	9	84	2268	5.947
		R	Inferior occipital gyrus	39	-78	-9			4.108
		R	Parahippocampal gyrus	33	-39	-9	106	2862	6.380
		R	Fusiform gyrus	36	-54	-12			5.703
		R	Lingual gyrus	30	-48	-9			5.539
		L	Parahippocampal gyrus	-27	-42	-9	76	2052	6.356
		L	Inferior temporal gyrus	-36	-39	-15			5.214
		L	Middle occipital gyrus	-33	-81	3	142	3834	5.969
Long > Brief	Natural objects > noise	L	Inferior occipital gyrus	-39	-87	-6	74	1998	6.384
		L	Middle occipital gyrus	-33	-93	12			5.903
Long > Brief	Building > noise	R	Middle occipital gyrus	33	-81	9	131	3537	5.581
		R	Parahippocampal gyrus	30	-39	-12			5.116
		R	Lingual gyrus	27	-60	-6			5.065
		R	Inferior occipital gyrus	33	-81	-9			4.533

		R	Fusiform gyrus	36	-54	-9			4.145
		L	Fusiform gyrus	-24	-48	-12	45	1215	6.606
		L	Inferior temporal gyrus	-39	-39	-15			4.910
Brief > Long	Human body > noise	R	Middle frontal gyrus	39	51	24	37	999	6.539

Supplementary Materials: Post hoc pairwise comparisons of subcategories

Supplementary Table S4. Post hoc pairwise comparisons of category information across the eight object subcategories.

This table reports post hoc pairwise comparisons following the repeated-measures ANOVA on category information across the eight object subcategories. Panel A shows the results for the Joint-ROI, Panel B shows the results for the Group-ROI, and Panel C shows the results for the Anatomical-ROI. Cell values indicate paired-samples *t*-statistics. Each comparison was computed as the category shown in the column header minus the category shown in the row header. Thus, positive values indicate greater category information for the column category, whereas negative values indicate greater category information for the row category. Significance levels were Bonferroni-corrected for multiple comparisons. $^{*}p < .05$, $^{**}p < .01$, $^{***}p < .001$.

Table S4A

	Human head	Human body	Animal face	Animal body	Tools	Large artifacts	Natural objects	Buildings
Human head	/							
Human body	3.551	/						
Animal face	3.948*	0.314	/					
Animal body	6.044***	1.360	1.127	/				
Tools	5.392**	2.810	2.818	2.479	/			
Large artifacts	6.620***	4.511**	3.963*	5.080**	1.656	/		
Natural objects	6.028***	3.123	3.199	3.096	-0.188	-2.532	/	
Buildings	4.470**	2.432	2.469	2.266	-0.038	-1.796	0.179	/

Table S4B

	Human head	Human body	Animal face	Animal body	Tools	Large artifacts	Natural objects	Buildings
Human head	/							
Human body	3.237	/						
Animal face	3.948*	-0.245	/					

Animal body	4.221*	0.131	0.369	/				
Tools	5.846***	2.565	3.013	3.030	/			
Large artifacts	8.524***	3.357	3.853*	5.931***	0.839	/		
Natural objects	6.227***	2.811	2.986	5.666***	0.266	-0.811	/	
Buildings	5.092**	1.541	1.822	2.353	-1.122	-2.401	-1.725	/

Table S4C

	Human head	Human body	Animal face	Animal body	Tools	Large artifacts	Natural objects	Buildings
Human head	/							
Human body	2.195	/						
Animal face	3.479	0.843	/					
Animal body	3.348	0.674	-0.139	/				
Tools	3.987*	2.446	2.015	2.402	/			
Large artifacts	5.295**	2.872	2.615	4.564**	0.655	/		
Natural objects	5.108**	3.144	2.875	4.711**	0.661	0.254	/	
Buildings	3.642	1.730	1.295	2.008	-0.392	-1.520	-1.662	/

Supplementary Results: Visual PC1 Control Analysis of Category Information

Model Specification:

Baseline model: CI ~ animacy_code + C(subj)

Visual control model: CI ~ animacy_code + scale(visual_pc1z) + C(subj)

CI denotes category information. Models were estimated using ordinary least squares (OLS) regression with subject fixed effects. Cluster-robust standard errors were clustered by subject. Animacy was effect-coded as animate = +0.5 and inanimate = −0.5. Therefore, the regression coefficient for animacy_code represents the animate–inanimate difference in category information.

Table S5A. Animacy-effect results from the baseline and visual-control models.

Analysis Dataset	ROI	Model	β	Cluster-robust *SE*	t	p	95% CI lower	95% CI upper	R^2	Adjusted R^2	AIC	BIC
Full set	Group-ROI	Baseline	0.11	0.02	5.19	< 0.001	0.07	0.15	0.34	0.25	-284.90	-228.47
Full set	Group-ROI	Visual Control	0.10	0.02	4.86	< 0.001	0.06	0.14	0.42	0.33	-299.73	-240.33
Full set	Joint-ROI	Baseline	0.17	0.04	4.58	< 0.001	0.09	0.24	0.36	0.27	-166.92	-110.49
Full set	Joint-ROI	Visual Control	0.16	0.04	4.38	< 0.001	0.09	0.23	0.41	0.32	-177.50	-118.10
Excluding human head	Group-ROI	Baseline	0.08	0.02	3.68	< 0.001	0.04	0.12	0.29	0.16	-279.52	-225.63
Excluding human head	Group-ROI	Visual Control	0.08	0.02	3.65	< 0.001	0.04	0.13	0.29	0.16	-277.94	-221.22
Excluding human head	Joint-ROI	Baseline	0.13	0.03	3.76	< 0.001	0.06	0.20	0.30	0.18	-166.29	-112.40
Excluding human head	Joint-ROI	Visual Control	0.13	0.04	3.77	< 0.001	0.06	0.20	0.30	0.17	-164.50	-107.78

Note: The full stimulus set included 144 observations from 18 subjects and 8 object subcategories. The analysis excluding the human-head category included 126 observations from 18 subjects and 7 object subcategories. β denotes the regression coefficient for animacy_code. Cluster-robust *SE*s were clustered by subject. The visual-control model included Visual homogeneity PC1 as an additional covariate.

Table S5B. Visual homogeneity PC1 results from the visual-control model.

Analysis Dataset	ROI	β	Cluster-robust SE	t	p	95% CI lower	95% CI upper	R^2	Adjusted R^2	AIC	BIC
Full set	Group-ROI	0.03	0.01	3.31	0.001	0.01	0.04	0.42	0.33	-299.73	-240.33
Full set	Joint-ROI	0.03	0.01	3.56	< 0.001	0.02	0.05	0.41	0.32	-177.50	-118.10
Excluding human head	Group-ROI	0.01	0.01	0.48	0.629	-0.02	0.03	0.29	0.16	-277.94	-221.22
Excluding human head	Joint-ROI	0.01	0.02	0.39	0.697	-0.02	0.04	0.30	0.17	-164.50	-107.78

Note: The full stimulus set included 144 observations from 18 subjects and 8 object subcategories. The analysis excluding the human-head category included 126 observations from 18 subjects and 7 object subcategories. β denotes the regression coefficient for Visual homogeneity PC1. Cluster-robust SEs were clustered by subject.
