# Supplementary material for: Animate Categories Show Higher Cross-Duration Representational Selectivity in Ventral Occipitotemporal Cortex Under Brief Visual Input
Source: Brain Sci. 2026 Jun 26;16(7):668. doi: 10.3390/brainsci16070668 (PMC13407226; doi:10.3390/brainsci16070668)
Supplement: Supplementary file 1 [file brainsci-16-00668-s001.zip › Supplementary Tables S6–S10- ROI Robustness_Subcategory Consistency_Top-N Sensitivity_and LOSO Stability.pdf]

Supplementary Tables: Subcategory CI Summary and Sensitivity Analysis of Animate Subcategories

Table S6. Descriptive statistics for cross-duration category information (CI) for each subcategory across three ROIs.

ROI	Category	Mean CI	*SD*	*SE*	95% CI
Joint-ROI	Human head	0.277	0.148	0.035	[0.203, 0.351]
	Human body	0.154	0.128	0.030	[0.090, 0.218]
	Animal face	0.143	0.136	0.032	[0.075, 0.211]
	Animal body	0.111	0.073	0.017	[0.074, 0.147]
	Tools	0.019	0.129	0.030	[-0.045, 0.083]
	Large artifacts	-0.033	0.076	0.018	[-0.071, 0.004]
	Natural objects	0.026	0.083	0.019	[-0.015, 0.067]
	Buildings	0.022	0.129	0.030	[-0.042, 0.086]
Group-ROI	Human head	0.192	0.102	0.024	[0.141, 0.243]
	Human body	0.086	0.096	0.023	[0.038, 0.134]
	Animal face	0.092	0.090	0.021	[0.047, 0.136]
	Animal body	0.083	0.047	0.011	[0.059, 0.106]
	Tools	0.007	0.080	0.019	[-0.033, 0.046]
	Large artifacts	-0.011	0.038	0.009	[-0.030, 0.008]
	Natural objects	-0.0001	0.057	0.013	[-0.029, 0.028]
	Buildings	0.033	0.074	0.017	[-0.003, 0.070]
Anatomical-ROI	Human head	0.115	0.084	0.020	[0.073, 0.157]
	Human body	0.062	0.073	0.017	[0.026, 0.098]
	Animal face	0.048	0.062	0.015	[0.017, 0.079]
	Animal body	0.050	0.041	0.010	[0.030, 0.071]
	Tools	0.005	0.070	0.016	[-0.030, 0.039]
	Large artifacts	-0.007	0.039	0.009	[-0.027, 0.012]
	Natural objects	-0.009	0.038	0.009	[-0.028, 0.010]
	Buildings	0.014	0.063	0.015	[-0.017, 0.046]

Note: Mean CI refers to the average category information, and 95% CI refers to the confidence interval.

Table S7. Statistical comparisons of each animate subcategory against the mean of all inanimate subcategories.

ROI	Animate Subcategory	Subcategory's Mean CI	Inanimate Mean CI	Mean Difference	*t* test	Cohen's d_z	95% CI	*p*
Joint-ROI	Human head	0.277	0.008	0.268	$t(17) = 6.05$	1.425	[0.175, 0.362]	< .001
	Human body	0.154	0.008	0.146	$t(17) = 3.60$	0.849	[0.060, 0.231]	0.004
	Animal face	0.143	0.008	0.134	$t(17) = 3.29$	0.774	[0.048, 0.221]	0.007
	Animal body	0.111	0.008	0.102	$t(17) = 3.80$	0.896	[0.045, 0.159]	0.003
Group-ROI	Human head	0.192	0.007	0.185	$t(17) = 6.91$	1.630	[0.128, 0.241]	< .001
	Human body	0.086	0.007	0.079	$t(17) = 2.76$	0.650	[0.019, 0.139]	0.015
	Animal face	0.092	0.007	0.084	$t(17) = 3.17$	0.747	[0.028, 0.141]	0.007
	Animal body	0.083	0.007	0.076	$t(17) = 5.03$	1.186	[0.044, 0.107]	< .001
Anatomical-ROI	Human head	0.115	0.001	0.115	$t(17) = 4.87$	1.148	[0.065, 0.164]	< .001
	Human body	0.062	0.001	0.061	$t(17) = 2.78$	0.655	[0.015, 0.108]	0.015
	Animal face	0.048	0.001	0.047	$t(17) = 2.42$	0.571	[0.006, 0.089]	0.027
	Animal body	0.050	0.001	0.050	$t(17) = 4.24$	1.001	[0.025, 0.074]	0.001

Note: The mean difference represents the CI of each animate subcategory minus the average signed CI of the four inanimate subcategories. All *p*-values were corrected for multiple comparisons using the FDR procedure.

Table S8. Sensitivity analysis of the animacy advantage after excluding one animate subcategory at a time.

ROI	Excluded animate subcategories	Mean difference	*t* test	Cohen's d_z	95% CI	*p*
Joint-ROI	Human head	0.127	$t(17) = 4.02$	0.948	[0.061, 0.194]	0.001
	Human body	0.168	$t(17) = 5.00$	1.178	[0.097, 0.239]	< .001
	Animal face	0.172	$t(17) = 5.14$	1.212	[0.102, 0.243]	< .001
	Animal body	0.183	$t(17) = 4.93$	1.162	[0.105, 0.261]	< .001
Group-ROI	Human head	0.080	$t(17) = 3.98$	0.939	[0.037, 0.122]	0.001
	Human body	0.115	$t(17) = 6.22$	1.466	[0.076, 0.154]	< .001
	Animal face	0.113	$t(17) = 6.23$	1.469	[0.075, 0.151]	< .001
	Animal body	0.116	$t(17) = 5.20$	1.226	[0.069, 0.163]	< .001
Anatomical-ROI	Human head	0.053	$t(17) = 3.40$	0.801	[0.020, 0.085]	0.003
	Human body	0.071	$t(17) = 4.53$	1.067	[0.038, 0.103]	< .001
	Animal face	0.075	$t(17) = 4.74$	1.118	[0.042, 0.109]	< .001
	Animal body	0.074	$t(17) = 4.06$	0.958	[0.036, 0.113]	0.001

Note: Each row shows the comparison between the mean CI of the remaining three animate subcategories and the mean CI of the four inanimate subcategories, after excluding the indicated animate subcategory. All *p*-values were corrected for multiple comparisons using the FDR procedure.

Supplementary Materials: Top-N Sensitivity and Leave-One-Subject-Out Stability Analyses

Table S9. Top-N sensitivity analysis of the animate-category advantage for the Joint-ROI.

Analysis set	Top-N	Mean difference	*t*-test	*p*	Cohen's *dz*	95% CI	Bootstrap 95% CI	Permutation *p*	LOSO direction
All categories	50	0.192	t(17) = 4.57	< 0.001	1.08	[0.104, 0.281]	[0.111, 0.271]	< 0.001	18/18
All categories	75	0.177	t(17) = 4.87	< 0.001	1.15	[0.100, 0.254]	[0.106, 0.244]	< 0.001	18/18
All categories	100	0.163	t(17) = 4.87	< 0.001	1.15	[0.092, 0.233]	[0.098, 0.224]	< 0.001	18/18
All categories	125	0.155	t(17) = 4.91	< 0.001	1.16	[0.088, 0.221]	[0.091, 0.213]	< 0.001	18/18
All categories	150	0.146	t(17) = 4.89	< 0.001	1.15	[0.083, 0.208]	[0.087, 0.201]	< 0.001	18/18
All categories	175	0.140	t(17) = 4.82	< 0.001	1.14	[0.079, 0.202]	[0.084, 0.194]	< 0.001	18/18
All categories	200	0.135	t(17) = 5.01	< 0.001	1.18	[0.078, 0.192]	[0.083, 0.185]	< 0.001	18/18
Excluding human head	50	0.149	t(17) = 3.80	0.001	0.90	[0.066, 0.231]	[0.074, 0.224]	0.001	18/18
Excluding human head	75	0.139	t(17) = 4.01	< 0.001	0.95	[0.066, 0.212]	[0.071, 0.203]	< 0.001	18/18
Excluding human head	100	0.127	t(17) = 4.02	< 0.001	0.95	[0.061, 0.194]	[0.067, 0.187]	0.001	18/18
Excluding human head	125	0.121	t(17) = 4.10	< 0.001	0.97	[0.059, 0.184]	[0.063, 0.176]	0.001	18/18
Excluding human head	150	0.114	t(17) = 4.13	< 0.001	0.97	[0.056, 0.172]	[0.062, 0.167]	< 0.001	18/18
Excluding human head	175	0.109	t(17) = 4.02	< 0.001	0.95	[0.052, 0.166]	[0.057, 0.159]	< 0.001	18/18
Excluding human head	200	0.105	t(17) = 4.19	< 0.001	0.99	[0.052, 0.157]	[0.056, 0.152]	< 0.001	18/18

Note: Mean difference indicates the category-information difference between animate and inanimate categories. The all-categories analysis included four animate and four inanimate

subcategories. The human-head-excluded analysis compared the remaining three animate subcategories with the four inanimate subcategories. Bootstrap confidence intervals were estimated at the subject level. Permutation p values were obtained from sign-flipping permutation tests. LOSO direction indicates the number of leave-one-subject-out iterations in which the animate advantage remained positive.

Table S10. Leave-one-subject-out stability analysis of the animate-category advantage.

ROI / mask	Analysis set	Full-sample mean	LOSO mean range	Positive iterations	Same-direction iterations
Anatomical-ROI	All categories	0.068	0.061-0.077	18/18	18/18
Anatomical-ROI	Excluding human head	0.053	0.045-0.061	18/18	18/18
Group-ROI	All categories	0.106	0.097-0.116	18/18	18/18
Group-ROI	Excluding human head	0.080	0.070-0.090	18/18	18/18
Joint-top50	All categories	0.192	0.173-0.218	18/18	18/18
Joint-top50	Excluding human head	0.149	0.128-0.172	18/18	18/18
Joint-top75	All categories	0.177	0.160-0.200	18/18	18/18
Joint-top75	Excluding human head	0.139	0.121-0.161	18/18	18/18
Joint-top100	All categories	0.163	0.148-0.183	18/18	18/18
Joint-top100	Excluding human head	0.127	0.110-0.147	18/18	18/18
Joint-top125	All categories	0.155	0.142-0.175	18/18	18/18
Joint-top125	Excluding human head	0.121	0.106-0.140	18/18	18/18
Joint-top150	All categories	0.146	0.131-0.165	18/18	18/18
Joint-top150	Excluding human head	0.114	0.098-0.131	18/18	18/18

Joint-top175	All categories	0.140	0.127-0.157	18/18	18/18
Joint-top175	Excluding human head	0.109	0.094-0.124	18/18	18/18
Joint-top200	All categories	0.135	0.123-0.150	18/18	18/18
Joint-top200	Excluding human head	0.105	0.093-0.119	18/18	18/18

Note: This table summarizes the leave-one-subject-out analysis of the main ROI definitions and the Joint-ROI top-N sensitivity analyses. In each iteration, one participant was excluded, and the animate-category advantage was recalculated using the remaining 17 participants. “Full-sample mean” indicates the animate-minus-inanimate category-information difference in the full sample. “LOSO mean range” indicates the minimum and maximum animate advantages across the 18 leave-one-subject-out iterations. “Positive iterations” indicates the number of iterations in which the animate advantage remained greater than zero. “Same-direction iterations” indicates the number of iterations in which the effect direction was consistent with the full-sample result.
